# Impacts of physical activity, sedentary behaviour, and sleep on depression symptoms in Canadian older adults 65 years of age and above: a compositional data analysis of the Canadian Longitudinal Study on Aging

**DOI:** 10.1186/s44167-024-00047-7

**Published:** 2024-03-08

**Authors:** Shawn Hakimi, Luc J. Martin, Mark W. Rosenberg

**Affiliations:** 1 School of Kinesiology and Health Studies, Queen’s University, 28 Division Street, Kingston, ON K7L 3N6 Canada; 2 Department of Geography and Planning, Queen’s University, Mackintosh-Corry Hall, 68 University Avenue, Kingston, ON K7L 3N6 Canada

**Keywords:** Ageing, Movement behaviour, Compositional, Mental health, Older adults, Time-use data, CLSA, Health promotion

## Abstract

**Background:**

Daily time spent in moderate-to-vigorous physical activity (MVPA), light-intensity physical activity (LIPA), sedentary behaviour (SB) and sleep (collectively known as ‘movement behaviours’) are compositional, co-dependent variables. However, most studies examining effects of these behaviours on depression outcomes in older adults do not account for this. Study objectives were to use compositional data analysis methods to (1) examine the relationship between movement behaviour composition (daily time spent in MVPA, LIPA, SB, sleep) and depression symptoms, (2) estimate the extent to which changing time spent in any given movement behaviour within the movement behaviour composition was associated with changes in depression symptoms.

**Methods:**

5643 older Canadian adults ≥ 65 years of age from the Canadian Longitudinal Study on Aging were studied using a quasi-longitudinal study design. Exposure was baseline daily movement behaviours; time spent in MVPA, LIPA and SB were derived from self-reported Physical Activity Scale for the Elderly responses. Night-time sleep was self-reported separately. Outcome was depression symptoms at follow-up obtained using the ten item Center for Epidemiologic Studies Depression Scale. Compositional data analysis was used to investigate associations between movement behaviours and depression symptoms.

**Results:**

Movement behaviour composition was significantly associated with depression symptoms. Time spent in MVPA [exp(B) = 0.97 (95% CI: 0.94, 0.99)] and sleep [exp(B) = 0.91 (95% CI: 0.85, 0.97)] relative to the remaining movement behaviours were associated with lower depression symptoms scores. Relative time spent in SB was associated with higher depression symptoms scores [exp(B) = 1.09 (95% CI: 1.04, 1.15)]. Time displacement estimates revealed that the greatest change in depression symptoms scores occurred when time spent in MVPA was decreased and replaced with LIPA, SB, sleep or combination of these behaviours (+ 0.22 to 0.26 points increase on depression symptoms scores for 30 min/day displacements).

**Conclusions:**

Daily movement behaviour composition was associated with depression symptoms in older Canadians. Replacing time in MVPA with equivalent time from any other behaviour was associated with increased depression symptoms. Preserving time spent in MVPA may play a key role in mitigating and improving mental health in this demographic.

**Supplementary Information:**

The online version contains supplementary material available at 10.1186/s44167-024-00047-7.

## Background

The world population is ageing rapidly; the number of adults aged 60 years and above is expected to double to two billion by 2050 [1]. Older people experience unique physical and mental health challenges that need to be recognised [1]. The ageing process is a complex interplay of physical, psychological, and social changes that are associated with increased morbidity and mortality, and in particular risk of mental health conditions [2]. Prevalence of mental health conditions are disproportionately higher in the older adult population, 15% of people aged 60 years and above suffer from a mental disorder, whereas it is around 10% in the overall population [1]. Specifically, depression occurs in 7% of this demographic and accounts for 5.7 years-lived with a disability. Today, ageing research is focussed on active healthy ageing (AHA) that emphasises the importance of health behaviours [3] to promote longevity and postponement of mental and physical decline, and abate economic and societal costs associated with ageing [4].

Movement behaviours, collectively physical activity (PA), sedentary behaviour (SB) and sleep, appear to be associated with depression symptoms in older adults, and are potentially modifiable factors through targeted interventions [5–8]. These behaviours encompass the changes in physical location or position throughout the day produced by the action of skeletal muscle and behaviours producing no specific movement (e.g., SB and sleep) which may interact to impact biological processes and physiological responses, and are classified by their energy expenditure demands on the body [e.g., metabolic equivalents of tasks (MET)] [9].

Moderate-to-vigorous physical activity (MVPA; e.g., brisk walking) levels are decreasing, and account for approximately 4% of waking time in adults [6, 10]. There is evidence that MVPA reduces risk for incident depression and symptoms; however, sufficient MVPA may not be as beneficial if SB remains high [11]. Light-intensity physical activity (LIPA; e.g., chores) levels have increased in recent years, and make up 25% of waking time [10] and is gaining traction as an important behaviour for older adults because it is easier to do than high intensity activity; however, evidence regarding its association with depression is inconsistent as moderate to low amounts may reduce risk, while high amounts increase it [12]. Increasing LIPA at the expense of MVPA may be detrimental, yet beneficial if it replaces SB [7, 10]. The above estimates are for the general adult population and are included to provide context about the proportion of the day spent in these behaviours, owing to a paucity in the literature of estimates for older adults specifically.

For older adults, sixty-percent of the waking day is made up of SB and levels are steadily increasing [10]. The evidence on SB is inconsistent and it may be that high levels (e.g., sitting) are an independent risk factor for depression symptoms [5]; however, associations regarding specific SB sub-types remain unclear. For example, active forms (e.g., reading, socialising) may reduce depression symptoms whereas passive forms (e.g., television) may increase them [13, 14]. SB can be reduced by increasing PA; however, most studies focus on MVPA which makes up a small portion of the day, and more extensive changes to daily movement behaviour patterns may be required to reduce risk, such as increasing LIPA.

Sleep duration remains steady in recent years with older adults spending on average seven hours sleeping per day [15, 16]. Consistent evidence shows that optimal sleep duration (~ 7 h/night) is important for mitigating depression risk, whereas low and high sleep durations increase it [8]. Achieving optimal sleep duration may not be straightforward owing to the complexities of sleep (e.g., sleep efficiency) and improving sleep quality may be more beneficial for older adults than increasing duration [17].

Research on movement behaviours as independent risk factors for depression outcomes in older adults is advancing [5–8]. However, inconsistent findings may be attributable to methodological limitations of previous work, such as cross-sectional designs and not accounting for the constrained nature of daily movement behaviours. Furthermore, many studies evaluate diagnosed depression as the outcome rather than depression symptoms; however, effects of depression in older adults may be underestimated as symptoms are often overlooked and untreated, because they occur on a continuum and commonly co-occur with other health problems [18]. Therefore, focussing on symptoms can help to elucidate the full extent of these associations, and improve practical application of findings to mental health promoting initiatives aimed at this demographic.

Of greater concern, most previous studies have not appropriately accounted for the co-dependence of movement behaviours which may obfuscate associations between movement behaviours and depression symptoms [19]. Time in a day is finite, and time spent in PA will displace time spent in at least one other behaviour, such as LIPA, SB or sleep. These behaviours have respective positive or negative effects on depression symptoms that must be considered when estimating effects of increasing or reducing time spent in one behaviour. However, standard statistical methods assume these behaviours are independent of each other, whereby increasing time in one will not influence time in the remaining behaviours. These limitations have been fully explained elsewhere [19–22]. To avoid them, novel methodologies that examine movement behaviours within a 24-h period are required [22]. Compositional data analysis (CoDA) is a statistical technique and set of principles for handling data that are relative components of a finite whole such as different activity periods within a day, that has recently been applied in the movement behaviour epidemiology field [19, 20]. CoDA allows for assessment of associations between a particular movement behaviour and depression symptoms while accounting for all other periods of the day (e.g., sleep), and estimating potential effects of reallocating time between behaviours.

The systematic review from Janssen et al. [23] was the first comprehensive assessment of CoDA studies in the field since inception of the first study in 2015 [20]. It showed that, at the time of publication (2020), there was one CoDA study examining mental health outcomes in older adults [24]. However, that study addressed self-rated mental health, and did not address depression symptoms specifically. Since then, three studies have used CoDA to examine depression outcomes in older adults, all with small sample sizes (n = 105; 1943; 1679) [25–27]. Olds et al. found that movement behaviour composition (i.e., time spent in MVPA + LIPA + SB + sleep) was significantly associated with depression symptoms, and that replacing sedentary time with PA and sleep reduced depression symptoms, respectively. Limitations of that study were time displacements were only presented graphically, so, readers cannot obtain specific values for time reallocation estimates, and confounder selection did not include diet or chronic disease measures.

The study from Hoffman et al. reported increasing MVPA with that time coming from SB or sleep had beneficial effects on depression symptoms, but not from LIPA which showed no significant association. Cabanas-Sanchez et al. reported that movement behaviour composition was significantly associated with depression symptoms, and relative time spent in MVPA was associated with lower symptoms. Time displacement estimates showed that replacing time spent in LIPA, SB and sleep with time in MVPA reduced depression symptoms and replacing time spent in LIPA with SB and sleep reduced symptoms, respectively. The authors also tested associations prospectively over a two-year follow-up and found that movement behaviour composition and time-replacement effects were not significantly associated with depression symptoms, except when replacing time spent in sleep with SB which reduced them.

Not accounting for how time is spent in the rest of the day may have contributed to inconsistent findings from previous studies not using a CoDA framework [5–8, 12–14, 28, 29]. For example, two meta-analyses found that SB was associated with higher risk of depression, but the association attenuated when adjusted for PA [5, 30]. Another meta-analysis found that PA reduced risk of depression; however, magnitude of the association differed by PA measure (e.g., frequency versus intensity) [6]. Estimating potential impact of replacing time in one movement behaviour for time in another on depression risk has important implications for health promotion initiatives because it can help determine which activities to promote or reduce.

There is a lack of studies using compositional methods to assess depression outcomes in older adults. To address previous limitations in the field, a compositional data analysis was conducted to: (1) investigate whether the composition of time spent in MVPA, LIPA, SB and sleep was associated with depression symptoms, and determine how respective movement behaviours are associated with depression symptoms while accounting for time spent in all other behaviours; (2) estimate the effects of replacing time in any given movement behaviour (MVPA, LIPA, SB or sleep) within the movement behaviour composition with other movement behaviours on depression symptoms.

## Methods

### Study design and participants

This study used data from the Canadian Longitudinal Study on Aging (CLSA) a nationally representative, stratified, random sample of 51,338 Canadian women and men aged 45–85 years at baseline (2015) who will be followed for at least 20 years (first follow-up 2018). The CLSA was established to collect information on biological, medical, psychological, social, lifestyle and economic aspects of Canadian older adults so that they can be studied to understand how they influence the ageing process [31]. The CLSA excludes residents of the Canadian territories, and some remote regions, persons on Federal First Nations reserves and other provincial First Nations settlements, full-time members of the Canadian armed forces, and institutionalised persons (including long-term care). Participants had to be able to complete interviews in English or French and be physically and cognitively able to participate on their own (e.g., able to hear, able to answer verbally). Three sampling frames were used for recruitment: (1) a subset of participants in Statistics Canada’s Canadian Community Health Survey-Healthy Aging; (2) provincial health care systems; (3) using random digit dialing of landline telephones. The CLSA has been further described elsewhere [32].

Our study used data from the CLSA comprehensive cohort (*n* = 30,097) which contains data collected via in-home interviews, questionnaires, and physical assessments completed at one of 11 CLSA data collection sites across Canada. Participants had to live within a 25-50 km radius of one of these sites and were recruited between 2012–2015. The full CLSA protocol was approved by various institutional ethics review boards across seven different provinces and is reviewed annually. Participants provided written, informed consent for their involvement.

**Fig. 1:**
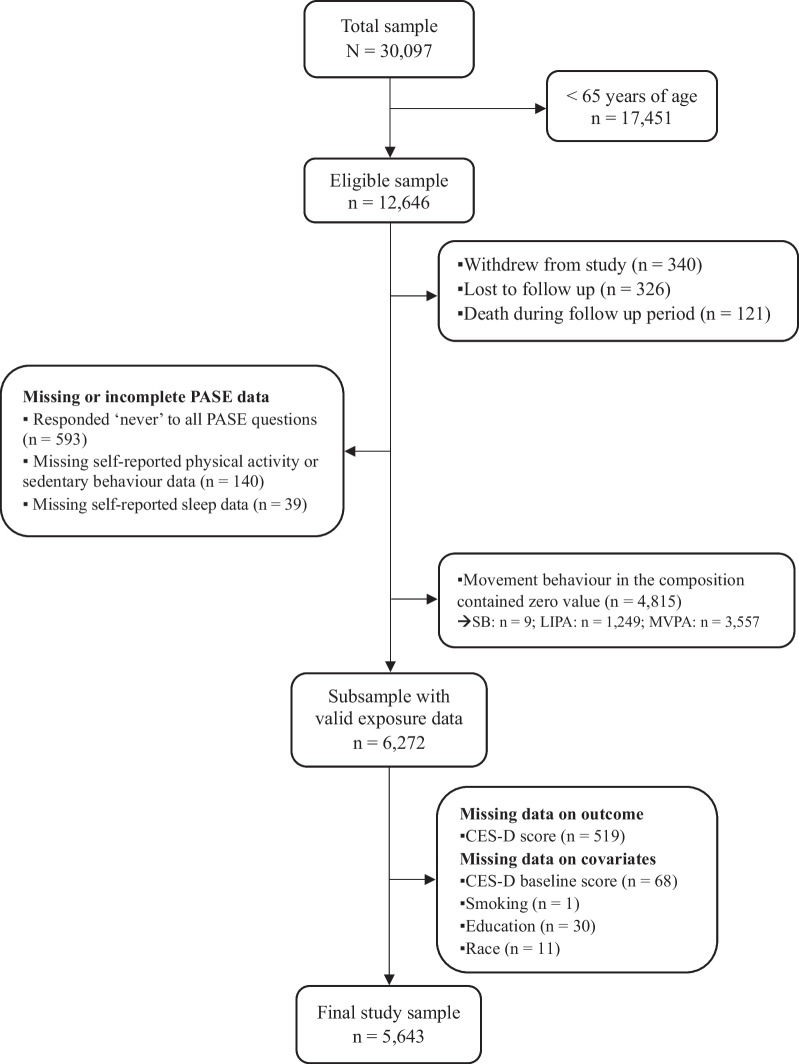
Flow chart of study participants. *CES-D* Center for Epidemiologic Studies Depression scale, *LIPA* light-intensity physical activity, *MVPA* moderate-to-vigorous physical activity, *PASE* Physical Activity Scale for the Elderly, *SB* sedentary behaviour

The final study sample for this project was made up of 5643 participants. Participants < 65 years of age, or with missing data for the exposure and covariate variables were excluded. A detailed flowchart summarising participant inclusion can be seen in Fig. 1. No statistically significant differences in age or sex were found when comparing the missing data group with the final study sample.

### Exposure: Daily movement behaviour composition

The exposure of interest was participants’ baseline (2015) daily movement behaviour composition, comprised of time spent in MVPA, LIPA, SB and sleep. All information regarding behaviours were self-reported by participants through a questionnaire administered by a CLSA staff member during an in-person at-home interview [33].

A modified version of the Physical Activity Scale for the Elderly (PASE) was used to collect information on MVPA, LIPA and SB for a seven-day period [34]. The PASE is a valid and reliable tool for measuring these behaviours in older adults and has been shown to have good test-rest reliability over a 3-to-7 week interval (Cronbach’s α = 0.75) and correlates well with accelerometry data [35]. Construct validity has also been established [34].

In relation to MVPA and LIPA, PASE gathers information on 70 different physical activities ranging from walking to strenuous exercise (e.g., calisthenics). Using established MET scores for older adults [36], activities were grouped into either MVPA (≥ 3.0 METs) or LIPA (< 3 METs) based on cut-offs for Canadian older adults ≥ 65 years of age [9]. For each activity, participants were asked about frequency and duration. For example, for walking, the questions were: “Over the past 7 days, how often did you take a walk outside your home or yard for any reason? For example, for pleasure or exercise, walking to work, walking the dog, etc.” and “On average, how many hours did you spend walking?” Frequency was recorded in categories of never (0 days), seldom (1 to 2 days), sometimes (3 to 4 days), and often (5 to 7 days). Duration was recorded in categories: less than 30 min, 30 min but less than 1 h, 1 h but less than 2 h, 2 h but less than 4 h, and 4 h or more. The frequency and duration categories derived from responses were used to estimate total weekly time in that activity using the PASE scoring manual hours per day conversion metric [34, 37]. Then, total hours were summed for all MVPA and LIPA activities, multiplied by 60 and divided by seven to derive total minutes per day, respectively.

In relation to SB, participants were asked the questions: “Over the past 7 days, how often did you participate in sitting activities such as reading, watching TV, computer activities or doing handicrafts?” and “On average, how many hours per day did you engage in these sitting activities?” The same frequency and duration category responses and PASE scoring manual conversion metric were used to estimate average weekly hours spent in SB. Total weekly hours were multiplied by 60 and divided by seven to derive total SB minutes per day.

Additionally, PASE asks about hours per week spent volunteering/working and the physical demands of the work being carried out grouped into four categories: (1) mainly sitting, (2) sitting, standing, light walking, (3) light-manual labour, and (4) heavy manual labour. Based on the category indicated, MET values were assigned for the type of work, and time was added to the total weekly hours of that movement behaviour. For example, if the participant indicated working 40 h per week, and selected category 1 (mainly sitting) for the physical demands of the work, 342.86-min (40 h × 60-min/7 days) were added to total daily time spent in SB.

For sleep, a single question from an 8-item questionnaire was used to obtain participant sleep time; the question was shown to have good reliability (Cronbach’s α = 0.83) [38]. Participants were asked: “During the past month, on average, how many hours of actual sleep did you get a night?” A whole integer was recorded. Total hours were multiplied by 60 to derive total sleep minutes per day.

Once total minutes per day for each behaviour were derived, they were summed and expressed as proportions of the daily movement behaviour composition. Participants who indicated zero values for any movement behaviour were excluded from the final study sample (Fig. 1).

### Outcome: depression symptoms

The outcome variable was depression symptoms at follow-up (2018). Depression symptoms was measured using the 10-item Center for Epidemiologic Studies Depression Scale (CES-D) which contains ten questions about feelings of depression, loneliness, hopefulness for the future, and restless sleep [39]. Participants were asked about frequency over the past seven days for each item measured with four possible options: [0 = rarely or never (< 1 day)], [1 = some of the time (1–2 days)], [2 = occasionally (3–4 days)], [3 = all the time (5–7 days)]. For example, “I was easily bothered by things that don’t usually bother me”. Total scores were obtained by summing the response values for each item [40]. Scores range from 0–30 with higher scores indicative of more depression symptoms. The CES-D has shown good validity and reliability as a screening tool for depression symptoms in older adults (Cronbach’s α = 0.80) [41]. We used continuous symptoms scores as the primary outcome to better represent the actuality of how depression symptoms develop on a continuum and maximise statistical power [42]. We also estimated possible cases of depression at follow-up using the established CES-D cut-off score for clinically relevant depression (scores ≥ 10). Imputation for up to three missing values was used by inputting the mean of the scores provided. This approach has been found to suitably account for missing values while maintaining validity of the CES-D [39, 43].

### Covariates

Covariates were investigated as possible confounders based on our understanding of the possible causal structure of the movement behaviour–depression symptoms relationship based on previous literature [5–8]. A directed acyclic graph (DAG) was used to depict causal associations between movement behaviours and depression symptoms and possible confounding variables (Additional file 1: Fig. S2). Based on the DAG, we identified confounding variables requiring statistical adjustment in our base models to block backdoor exposure-outcome pathways and improve estimate accuracy of the direct effect of movement behaviours on depression symptoms.

The confounding variables included in our analysis were: *age* (continuous, years) determined by date of birth information provided by participants. *Sex* was recorded as male or female. For *race*, participants indicated their racial background from the following categories: White, Black, Korean, Filipino, Japanese, Chinese, South Asian, Arab, West Asian, Latin American, other racial origin (only), multiple racial origins. These were categorised into white/non-white, as most of the sample was white. For *employment status*, participants were asked “During the past 7 days, did you work for pay or as a volunteer? Yes/No”, responses were categorised as ‘employed/volunteer’ and ‘unemployed’ [34]. For *marital status*, participants were asked “What is your current/marital/partner status?”, responses were categorised as single, married/common law, widowed, divorced/separated. *Smoking status* was determined by the question “What is your smoking status?”, categorised as never, former smoker, current smoker. For *education level*, participants were asked four questions regarding highest level of education achieved. Responses were categorised into < secondary school, secondary school graduation (no post-secondary education), some post-secondary education, or post-secondary degree/diploma. For *alcohol consumption*, participants provided information on number of drinks consumed of beer, red wine, white wine, liquor or spirit, or another kind of alcohol, inclusively during a typical week from the past 12 months on weekdays and weekends, respectively. Total weekday and weekend drinks were summed to derive total drinks per week and divided by seven to obtain average daily number of drinks [44], and categorised into non-drinkers (zero drinks consumed), light drinkers (≤ 1 drink per day for females, ≤ 2 for males), moderate drinkers (≤ 3 drinks per day for females, ≤ 4 for males), and heavy drinkers (> 3 drinks per day for females, > 4 for males) based on existing guidelines [45, 46]. A *diet quality score* was derived for each participant based on the Prospective Urban Rural Epidemiological (PURE) dietary quality score, a validated healthy diet measure comprised of seven key food groups shown to be associated with lower mortality and better health: fruits, vegetables, nuts, legumes, fish, dairy, meats (chicken and red meat) [47]. Information on habitual food intake over the past 12 months of specific items captured by the validated CLSA Short Diet Questionnaire were used to derive daily frequency of consumption of the seven food groups. [48]. Following PURE recommendations, daily frequency of consumption for each food group was divided into quintiles, with a value of one assigned to the lowest quintile and five for the highest. The value assigned to each quintile for each food group was summed resulting in scores ranging from seven (worst diet) to 35 (best diet). Final scores were divided into quintiles for the analysis [49]. A *comorbidity burden score* was derived to assess 35 International Classification of Diseases (tenth edition) conditions, as adults ≥ 65 years are known to have co-occurrence of multiple medical conditions [50]. Information on chronic conditions was captured using the self-report question stem “has a doctor ever told you that you have…’ and confirming that the participant had the condition for at least the past six months. To obtain scores, total number of chronic conditions was multiplied by an age-adjustment factor developed for use with CLSA data to account for interaction between sum of chronic conditions and age [51]. Our a priori DAG indicated that adjustment for baseline depression symptoms was necessary to estimate the causal associations between movement behaviours and depression symptoms. Baseline depression symptoms was measured with the same CES-D scale described previously [39].

We did not adjust for BMI or fitness level because the DAG showed that the former may be on the causal pathway between movement behaviour and depression symptoms and that the latter is a mediator. Our DAG also indicated family history/genetics as a potential confounder; however, this information was not available in our dataset.

Full standard operating procedures and questions for all variables analysed can be found on the CLSA website, and in the study protocol [52, 53].

### Analysis strategy

Statistical analyses were performed using SPSS version 29 (IBM Corp, Armonk, NY). Initial power analysis was carried out to estimate power of our study sample to detect small, medium, and large effect sizes with α set at 0.05 (Additional file 1: Fig. S1). CLSA analytical weights were not applied, as we tested our base models with both weighted and unweighted data and found no considerable difference in the estimated effects. Recent studies using CLSA data have also observed this, and concluded that the unweighted regression methods are no different than weighted [54, 55]. To establish temporality and avoid some of the limitations of cross-sectional analyses a quasi-longitudinal study design was used to model the associations between baseline movement behaviour composition (2015) and follow-up depression symptoms (2018). We call this quasi-longitudinal because we did not go beyond identifying changes between the two time points and the methods we used address changes in the parameter estimates between the two time points only.

Conventional descriptive statistics were used to describe the study sample. CoDA was used to describe movement behaviour variables, determine their co-dependence, and assess the association between movement behaviour composition and its components with depression symptoms (aim 1), and estimate the effects of replacing time spent in one movement behaviour with time spent in another on future depression symptoms (aim 2).

CoDA is appropriate for data that make up proportions of a finite whole (i.e., movement behaviours in a 24-h day). Detailed descriptions of how to use CoDA to analyse movement behaviour data is available elsewhere; therefore, a brief description is provided below [19, 20]. Geometric means for the proportion of time spent in each movement behaviour were calculated, as they better represent the central tendency of compositional data than the arithmetic mean. The codependence between movement behaviours can then be assessed using pair-wise log ratio variances between all behaviours [(e.g., variance of ln (SB/MVPA)] and scaled to aid in interpretation ${[(e}^{- \frac{{t}^{2}}{2}})$ where* t* is any log-ratio variance]. Values for pair-wise log-ratio variances range from zero to one, with values closer to one indicating higher codependence. These values were then represented in a variation matrix.

Prior to fitting the regression models, movement behaviour variables were transformed from their natural space, the constrained simplex S^d^ (i.e., a 24-h day) onto standard real space where standard statistical techniques can be used. For this step, *isometric log ratios (ilr)* were used to express the movement behaviour composition as ratios of its parts (i.e., absolute time spent in MVPA, LIPA, SB, sleep). Sequential binary partitioning was used to determine the appropriate configuration of *ilr* coordinates for each movement behaviour, so that each one was assessed as the main behaviour in relation to the remaining movement behaviours (e.g., time spent in MVPA *relative* to LIPA, SB and sleep) [56, 57]. Daily movement behaviour composition allotted into four parts (MVPA, LIPA, SB, sleep) was expressed as three *ilr* coordinates that capture the combined distribution of all parts of the composition. For example, *ilr* coordinates for MVPA relative contribution are written as:

$${z}_{i1}=\sqrt{\frac{3}{4}} {\text{ln}}\left(\frac{{MVPA}_{i}}{\sqrt[3]{{{LIPA}_{i} x SB}_{i} x {sleep}_{i}}}\right) {z}_{i2}= \sqrt{\frac{2}{3}} {\text{ln}}\left(\frac{{LIPA}_{i}}{\sqrt[2]{{sleep}_{i} x {SB}_{i}}}\right) {z}_{i3}=\sqrt{\frac{1}{2}} \left(\left.\frac{{sleep}_{i}}{{SB}_{i}}\right)\right.$$

Then, regression models fitted to CES-D scores were constructed using the corresponding set of three *ilr* coordinates for each movement behaviour along with confounding variables as explanatory variables. Four models were constructed, one for each behaviour. Negative binomial regression was used due to the right skew and overdispersion of CES-D scores. CES-D is scored only as a positive integer or zero value (zero is considered the best score). The negative binomial model is well suited to handle discrete values and expected zeros in the outcome variable. Details from preliminary testing carried out to assess the dataset for necessary criteria to employ negative binomial regression are provided in Additional file 1: Fig. S3.

Overall maximum likelihood test statistics from robust regression models were used to determine significance of the entire movement behaviour composition (aim 1). The regression coefficient and *p*-value corresponding to the first *ilr* coordinate variable (*z*_*i1*_) were used to determine if that specific movement behaviour was significantly associated with depression symptoms *relative* to time spent in the remaining movement behaviours (aim 1). Only the first *ilr* coordinate variable in each model was interpreted as it contains all relevant information regarding a participant’s movement behaviour composition (e.g., MVPA relative to LIPA, SB, sleep). The second and third *ilr* coordinates are used to fit the model, but not meaningfully interpreted.

The base models parameter estimates are difficult to interpret as change in CES-D scores associated with time spent in a specific behaviour without back transformation [20]. To present those results in a more meaningful way we used model coefficients to conduct compositional isotemporal substitutions to estimate the extent to which displacing time spent in one movement behaviour with one or more of the remaining behaviours predict change in depression symptoms (aim 2). The base model coefficients represent the estimated effect on depression symptoms scores when the behaviour in the numerator changes relative to the geometric mean of all the other behaviours (denominator). Since compositional data are relative, time displacement predictions must be made in relation to a reference point; to that end the mean movement behaviour composition from the study sample was used [56].

Compositional isotemporal modelling allows for simulation of different scenarios such that hypothetical time substitutions (e.g., reducing SB and increasing MVPA) can be modelled to estimate possible effect on depression. Time substitutions are considered hypothetical because they are based on simulations rather than actual changes in the movement behaviour data. We carried out initial time displacements for 30-min/day to align with previous CoDA studies [26, 27, 58] and national movement behaviour guidelines for older adults [59]. Additional estimates ranging from 15 to 120 min/day were completed, and graphically for up to ± 2 standard deviations (SD) of the mean for each behaviour. Theoretically, it is possible to estimate replacements larger than this (any amount up to the limit of the movement behaviour composition being analysed); however, substantive changes in the movement behaviour patterns in the study population are less plausible. Time substitution estimates represent theoretical points change in depression symptoms scores. We also ran logistic regression models with a dichotomised outcome variable indicating new cases of depression (scores ≥ 10). Fully adjusted models included all confounding variables described above.

### Sensitivity analyses

A priori planned sensitivity analyses were conducted to test the robustness of our findings and evaluate alternative explanations: 1) Effect modification*:* statistical interactions by sex and age were investigated based on their a priori consideration as effect modifiers. We examined whether sex and age (median split, < 71 and ≥ 71 years) modified associations by adding interaction terms “effect modifier * *z*_*i*1_”, “effect modifier * *z*_*i*2_” and “effect modifier * *z*_*i*3_” with each movement behaviour. 2) We repeated the main analysis excluding participants with diagnosis of clinical depression to reduce the risk of reverse causation. 3) Following STROBE guidelines [60] we re-ran base models randomly removing 10% of the cases and checking for a significant deviation in the results. 4) We calculated e-values for the main findings to estimate potential bias from unmeasured and residual confounding. The e-value is an estimate of the strength of an unmeasured confounder variable that would be required to nullify the observed association between our exposure and outcome while accounting for all measured covariates [61].

## Results

### Descriptive characteristics

Descriptive baseline sociodemographic and health behaviour characteristics of the study sample are presented in Table 1. For details on participant eligibility in the study sample and missing data for each variable of interest see Fig. 1. On average participants were 72.40 years of age. The distribution of males (51.9%) was higher than females (48.1%). The majority were white (96.1%), non-smokers or former smokers (96%), non- or light- drinkers (80.2%), married or common-law (65.9%), unemployed (55.7%). Mean CES-D score was 4.68 and 613 (10.9%) participants met the CES-D threshold for depression at follow-up.

**Table 1:** Participant characteristics at baseline

	Full sample (n = 5643)		Males (n = 2931)		Females (n = 2712)	
Variable	n	%	n	%	n	%
Age, mean (SD)	72.40 (5.51)	–	72.39 (5.52)	–	72.41 (5.49)	–
Race						
Non-white	220	3.9	142	4.8	78	2.9
White	5423	96.1	2789	95.2	2634	97.1
Smoking status						
Never	2535	44.9	1146	39.1	1389	51.2
Former	2880	51.0	1643	56.1	1237	45.6
Current	228	4.1	142	4.8	86	3.2
Alcohol consumption						
Heavy drinker	116	2.1	79	2.7	37	1.4
Moderate drinker	1000	17.7	411	14.0	589	21.7
Light drinker	2855	50.6	1746	59.6	1,109	40.9
Non-drinker	1672	29.6	695	23.7	977	36.0
Diet quality score^a^						
Quintile 1 (worst)	1203	21.3	737	25.2	466	17.2
Quintile 2	1228	21.8	668	22.8	560	20.7
Quintile 3	924	16.4	451	15.4	473	17.4
Quintile 4	1309	23.2	646	22.0	663	24.4
Quintile 5 (best)	979	17.3	429	14.6	550	20.3
Marital status						
Single	307	5.4	111	3.8	196	7.3
Divorced/separated	715	12.7	248	8.5	467	17.2
Widowed	904	16.0	231	7.9	673	24.8
Married/common law	3717	65.9	2341	79.8	1376	50.7
BMI, mean (SD)	27.27 (4.62)	–	27.42 (3.98)	–	27.10 (5.21)	–
Employment						
Unemployed	3143	55.7	1592	54.3	1551	57.2
Employed	2500	44.3	1339	45.7	1161	42.8
Comorbidity burden score^b^, mean (SD)	1.45 (0.76)	–	1.30 (0.69)	–	1.61 (0.79)	–
Baseline CES-D score, mean (SD)	4.59 (4.04)	–	4.01 (3.65)	–	5.22 (4.35)	–

*SD* standard deviation, *BMI* body mass index, *CES-D* Centre for Epidemiologic Studies Depression Scale

^a^ Diet score indicates low consumption on seven healthy food categories, scores range from 7–35, and are divided into quintiles. Score was calculated using the Prospective Urban Rural Epidemiological (PURE) diet quality score or “PURE healthy diet score”. ^b^ Total number of chronic diseases (35 ICD-10 chronic diseases) multiplied by age adjustment factor

Geometric and arithmetic means for MVPA, LIPA, SB and sleep (%, hours:minutes per day) are presented in Table 2; sex stratifications are presented in Additional file 1: Table S1. On average, MVPA, LIPA, SB and sleep accounted for 4% [36 min], 20% (2 h:49 min), 28% (3 h:54 min) and 48% (6 h:47 min) of the movement behaviour composition, respectively. Males spent more time in MVPA (5%, 42 min) and LIPA (21%, 3 h:03 min) than females (4%, 31 min; 19%, 2 h:36 min). Table 3 shows the variation matrix of pair-wise log ratio variances, sex stratification matrices are presented in Additional file 1: Table S2. The greatest codependence was between SB and LIPA (0.99), the lowest was between MVPA and LIPA (0.43). Therefore, MVPA had the lowest co-dependence with the other movement behaviours.

**Table 2:** Compositional (geometric) and arithmetic means of time spent in MVPA, LIPA, SB, and sleep per day

	Compositional		Arithmetic	
Movement behaviour	Mean (hours:minutes)	Proportion	Mean (hours:minutes ± SD)	Proportion
MVPA	0:36	0.04	0:55 (± 1:01)	0.06
LIPA	2:49	0.20	3:22 (± 2:13)	0.22
SB	3:54	0.28	4:17 (± 1:38)	0.28
Sleep	6:47	0.48	6:54 (± 1:11)	0.44

*SD* standard deviation

**Table 3:** Compositional variation matrix of time spent in MVPA, LIPA, SB, and sleep

	MVPA	LIPA	SB	Sleep
MVPA	0	0.43	0.45	0.65
LIPA	0.43	0	0.99	0.93
SB	0.45	0.99	0	0.90
Sleep	0.65	0.93	0.90	0

### Model results

Fully adjusted estimates for depression symptoms with each movement behaviour relative to the remaining movement behaviours are presented in Table 4; sex stratifications are presented in Additional file 1: Table S3. Movement behaviour composition was significantly associated with depression symptoms (*p* < 0.001 for overall model fit). Base model regression coefficients can be interpreted as proportional change in depression symptoms associated with an increase in time spent in that movement behaviour relative to the time spent in the remaining behaviours. Relative time spent in MVPA [exp(B) = 0.97, 95% CI: 0.94, 0.99, *p* = 0.02] and sleep [exp(B) = 0.91, 95% CI: 0.85, 0.97, *p* = 0.005] were associated with lower depression symptoms. Relative time spent in SB was associated with higher depression symptoms [exp(B) = 1.09, 95% CI: 1.04, 1.15, *p* < 0.001]. Relative time spent in LIPA was not associated with depression symptoms [exp(B) = 1.04, 95% CI: 0.99, 1.08, *p* = 0.08].

**Table 4:** Compositional negative binomial regression model estimates for depression symptoms

	Model	MVPA	*p*-value	LIPA	*p*-value	SB	*p*-value	Sleep	*p-*value
	*p*-value	exp($\gamma$)* (95% CI)		exp($\gamma$)* (95% CI)		exp($\gamma$)* (95% CI)		exp($\gamma$)* (95% CI)	
Unadjusted	p < 0.001	0.92 (0.90, 0.95)	< 0.001	0.98 (0.93, 1.02)	0.32	1.27 (1.21, 1.34)	< 0.001	0.80 (0.72, 0.90)	< 0.001
Adjusted	p < 0.001	0.97 (0.94, 0.99)	0.02	1.04 (0.99, 1.08)	0.08	1.09 (1.04, 1.15)	< 0.001	0.91 (0.85, 0.97)	0.005

Adjusted for age, sex, race, employment status, education level, marital status, smoking status, alcohol consumption, diet quality score, comorbidity burden score, baseline depression symptoms. *Exponential of regression coefficient ($\upgamma$). These values represent the proportional unit change in depression symptoms score per unit increase in the associated *ilr* coordinate, as time allocated to the movement behaviour in the numerator against the geometric mean of the others in the denominator. In these models, only *z*_1_ is interpretable as it contains the relative information for all 24 h movement behaviours (i.e., MVPA, LIPA, SB and sleep). The p-value indicates a statistically significant association between daily movement behaviour and depression symptoms score after accounting for the time spent in the remaining behaviours

### Compositional isotemporal substitution modeling

**Fig. 2:**
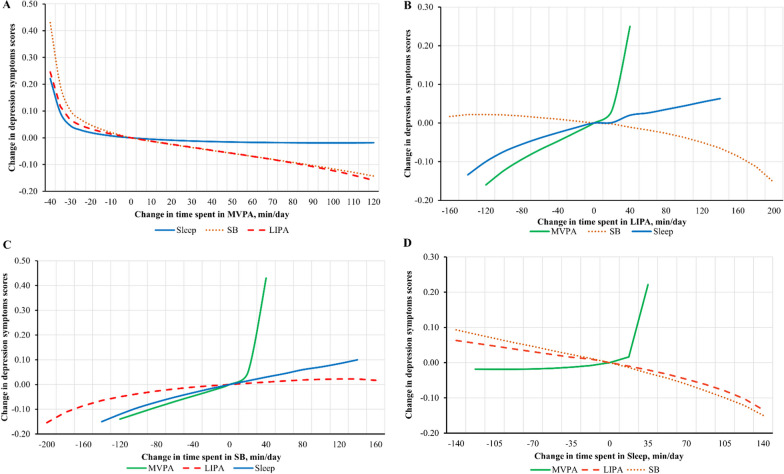
**a**–**d** Estimates for changes in depression symptoms scores associated with hypothetical time displacements from one movement behaviour to another. All estimates adjusted for age, sex, race, employment status, education level, marital status, smoking status, alcohol consumption, diet quality score, comorbidity burden score, baseline depression symptoms. Estimates reflect the hypothetical change in depression symptoms scores associated with reallocating time spent in each movement behaviour based on parameter estimates from compositional regression. The difference in minutes/day are modelled around the mean movement behaviour composition (reference). Time is substituted between the movement behaviour on the x-axis and the movement behaviour indicated by the line. For example, Panel B shows estimated scores associated with hypothetically changing the mean amount of time spent in LIPA. As more minutes are added to LIPA, it is estimated that scores will increase if this time is taken from MVPA but decrease if this time is added to sleep. Substitutions were not made beyond the range of ± two SD for the mean of each movement behaviour (e.g., no more than 120-min per day were added to the mean 36-min per day spent in MVPA)

**Fig. 3:**
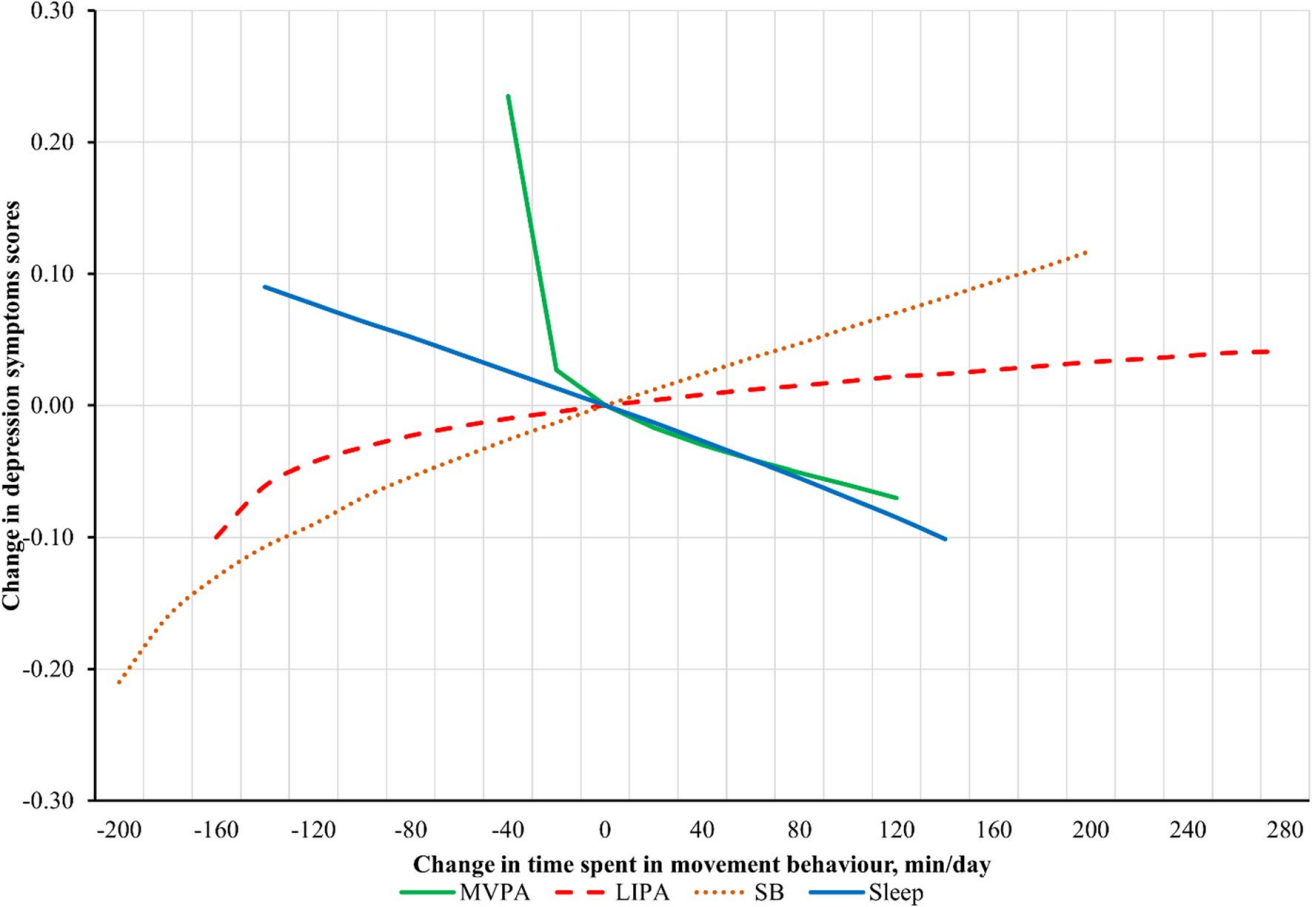
Estimates for changes in depression symptoms scores associated with hypothetical time displacements between one and the remaining movement behaviours proportionally. All estimates adjusted for age, sex, race, employment status, education level, marital status, smoking status, alcohol consumption, diet quality score, comorbidity burden score, baseline depression symptoms. Estimates reflect the hypothetical change in depression symptoms scores associated with reallocating time between one movement behaviour and the remaining behaviours based on parameter estimates from compositional regression. The difference in minutes/day are modelled around the mean movement behaviour composition (reference). For example, if more time is hypothetically allocated to sleep and removed from the remaining movement behaviours, the estimated depression symptoms scores decrease. Substitutions were not made beyond the range of ± two standard deviations for the mean of each movement behaviour (e.g., no more than 120-min per day were added to the mean 36-min per day spent in MVPA)

Figures 2 and 3, and Additional file 2: Figs. S1-S4 for sex stratifications depict how parameter estimates from the base models were used to estimate change in depression symptoms scores associated with equivalent time displacements from the mean movement behaviour composition (reference point). Figure 2 and Table 5 shows predicted changes in depression symptoms with replacing time spent in one movement behaviour with another behaviour (e.g., removing 30-min/day of MVPA and replacing it with 30-min/day of SB). Figure 3 and Table 6 show estimated changes in depression symptoms associated with displacing time spent in one movement behaviour to or from a combination of behaviours proportionally based on the geometric mean of each behaviour. For example, when 30-min/day was removed from mean time spent in MVPA that amount of time was proportionally redistributed to the remaining movement behaviours such that 6.25, 8.75 and 15 min were added to LIPA, SB, and sleep, respectively. Additional estimates for time displacements ranging from 15–120 min/day for the full sample and sex stratifications are presented in Additional file 2: Tables S1-S5.

**Table 5:** Estimated change in depression symptoms as measured by CES-D score scale that would occur by displacing 30-min between movement behaviours

Remove 30-min per day from		Add 30-min per day to		
	MVPA	LIPA	SB	Sleep
MVPA	–	0.25 (0.15, 0.34)	0.25 (0.15, 0.34)	0.22 (0.13, 0.32)
LIPA	− 0.02 (− 0.11, 0.08)	–	0.01 (− 0.09, 0.10)	− 0.01 (− 0.11, 0.08)
SB	− 0.04 (− 0.14, 0.05)	− 0.01 (− 0.10, 0.08)	–	− 0.02 (− 0.11, 0.07)
Sleep	0.01 (− 0.09, 0.10)	0.02 (− 0.08, 0.11)	0.02 (− 0.07, 0.12)	–

Data presented as estimated points change in CES-D score (95% confidence interval). All estimates adjusted for age, sex, race, employment status, education level, marital status, smoking status, alcohol consumption, diet quality score, comorbidity burden score, baseline depression symptoms. Values reflect estimated points change in CES-D score with re-allocating 30-min from the movement behaviour in the column to the movement behaviour in the row using the mean movement behaviour composition as the reference. For example, replacing 30-min of MVPA with 30-min of LIPA would result in an increase of 0.25 points in depression symptoms. Lower CES-D score indicates less depression symptoms, higher score indicates more depression symptoms. MVPA moderate-to-vigorous physical activity, LIPA light-intensity physical activity, SB sedentary behaviour

**Table 6:** Estimated change in depression symptoms as measured by CES-D score scale that would occur by displacing 30-min from one movement behaviour to remaining behaviours proportionally

Movement behaviour	Remove 30-min of column behaviour and add to remaining behaviours	Add 30-min to column behaviour from remaining two behaviours
MVPA	0.23 (0.14, 0.33)	– 0.02 (– 0.12, 0.07)
LIPA	− 0.01 (− 0.10, 0.09)	0.01 (− 0.09, 0.10)
SB	− 0.02 (− 0.11, 0.07)	0.02 (− 0.08, 0.11)
Sleep	0.02 (− 0.07, 0.11)	− 0.02 (− 0.11, 0.07)

Data presented as estimated points change in CES-D score (95% confidence interval). All estimates adjusted for age, sex, race, employment status, education level, marital status, smoking status, alcohol consumption, diet quality score, comorbidity burden score, baseline depression symptoms. Values reflect estimated points change in CES-D score with re-allocating time from one behaviour to the remaining movement behaviours proportionally (or vice versa) using the mean movement behaviour composition as the reference. For example, replacing 30-min of MVPA with 6.25-min LIPA, 8.75-min SB and 15-min sleep would result in an increase of 0.23 points in depression symptoms. Lower CES-D score indicates less depression symptoms, higher score indicates more depression symptoms. MVPA moderate-to-vigorous physical activity, LIPA light-intensity physical activity, SB sedentary behaviour

Estimates revealed increased depression symptoms scores with reallocating 30-min/day of MVPA into LIPA (0.25, 95% CI: 0.15, 0.34), SB (0.25, 95% CI: 0.15, 0.34), sleep (0.22, 95% CI: 0.13, 0.32), and combination of remaining behaviours proportionately (0.23, 95% CI: 0.14, 0.33).

In logistic regression models, replacing 30-min/day of MVPA with 30-min/day of LIPA (OR = 1.14, 95% CI: 1.13, 1.15), SB (OR = 1.17, 95% CI: 1.16, 1.18), sleep (OR = 1.07, 95% CI: 1.06, 1.08) and combination (OR = 1.12, 95% CI: 1.11, 1.13) was associated with higher odds of depression. Conversely, adding 30-min/day to MVPA from LIPA (OR = 0.93, 95% CI: 0.92, 0.94), SB (OR = 0.85, 95% CI: 0.84, 0.86), sleep (OR = 0.99, 95% CI: 0.98, 1.00) and combination (OR = 0.95, 95% CI: 0.94, 0.96) was associated with lower odds of depression. For LIPA, replacing 30-min/day with MVPA (OR = 0.93, 95% CI: 0.92, 0.94) and sleep (OR = 0.95, 95% CI: 0.94, 0.96) was associated with lower odds of depression, and higher odds when that time was put into SB (OR = 1.06, 95% CI: 1.05, 1.07) and combination of remaining behaviours (OR = 1.01, 95% CI: 1.00, 1.02). Adding 30-min/day to LIPA from MVPA (OR = 1.14, 95% CI: 1.13, 1.15) and sleep (OR = 1.03, 95% CI: 1.02, 1.04) was associated with higher odds for depression, and lower odds when that time came from SB (OR = 0.94, 95% CI: 0.93, 0.95) and combination of remaining behaviours (0.99, 95% CI: 0.98, 1.00).

Replacing 30-min/day of SB with MVPA (OR = 0.85, 95% CI: 0.84, 0.86), LIPA (OR = 0.94, 95% CI: 0.93, 0.96), sleep (OR = 0.90, 95% CI: 0.89, 0.91), or combination of remaining behaviours (OR = 0.92, 95% CI: 0.91, 0.93) was associated with lower odds of depression. Increasing SB by 30-min/day with time from MVPA (OR = 1.17, 95% CI: 1.16, 1.18), LIPA (OR = 95% CI: 1.05, 1.07), sleep (OR = 1.09, 95% CI: 1.08, 1.10) or combination of remaining behaviours (OR = 1.09, 95% CI: 1.07, 1.09) was associated with higher odds of depression.

For sleep, replacing 30-min/day with MVPA (OR = 0.99, 95% CI: 0.98, 1.00), LIPA (OR = 1.03, 95% CI: 1.02, 1.04), SB (OR = 1.09, 95% CI: 1.08, 1.10) or combination of remaining behaviours (OR = 1.08, 95% CI: 1.07, 1.09) was associated with higher odds of depression. Conversely, adding 30-min/day to sleep from MVPA (OR = 1.07, 95% CI: 1.06, 1.08) was associated with higher odds of depression, and lower odds when that time came from LIPA (OR = 0.95, 95% CI: 0.94, 0.96), SB (OR = 0.90, 95% CI: 0.89, 0.91) or combination of remaining behaviours (OR = 0.93, 95% CI: 0.92, 0.94). Logistic regression results for the full sample and sex stratifications can be seen in Additional file 2: Tables S6-S11.

Time reallocation estimates suggest that the relationship between changes in movement behaviours and depression symptoms are asymmetrical (Figs. 2 and 3; Additional file 1: Fig. S1-S4). For instance, the magnitude of effect for reallocating 30-min of MVPA into remaining behaviours was larger (0.23, 95% CI: 0.14, 0.33) than the inverse reallocation of 30-min from the remaining behaviours into MVPA (– 0.02, 95% CI: – 0.12, 0.07).

### Sensitivity analyses results

There was evidence of effect modification for sex. Interaction terms containing MVPA (*p* = 0.006), LIPA (*p* = 0.05), and sleep (*p* < 0.001), relative to remaining movement behaviours were statistically significant. Thus, analyses were conducted on the full sample, and stratified by sex. For age, only the interaction term containing sleep relative to the remaining movement behaviours was statistically significant (*p* = 0.008). Therefore, we did not stratify by age group because this difference in the strength of the association may be due to variation in MVPA levels, rather than differential association between age groups.

When participants with diagnosed clinical depression were excluded and we reran the fully adjusted models in a sample of 5030 with complete data (89.1% of our sub sample), results were largely consistent with our main analysis. One difference was magnitude of the associations for substituting time out of MVPA and into any of the other movement behaviours were attenuated (Additional file 1: Tables S4 and S6).

After randomly removing 10% of the study sample and rerunning the fully adjusted models in a sample of 5094 with complete data, results were consistent with our main analysis (Additional file 1: Tables S5 and S7) [60].

The e-values show our main findings are unlikely to be nullified by an unmeasured confounding variable. One exception was for the effects of substituting time out of MVPA and into sleep which produced an e-value < 1: Results S1.

## Discussion

This study is the first to use CoDA to examine associations between daily movement behaviours and depression symptoms in the ≥ 65 years of age population. We found that baseline daily movement behaviour composition was associated with follow-up depression symptoms. Relative time spent in MVPA and sleep were significantly associated with lower depression symptoms scores, whereas time spent in SB was associated with higher scores. Theoretically replacing time spent in MVPA with LIPA, SB, sleep or combination of these behaviours was associated with higher depression symptoms scores, while adding time to MVPA from SB was associated with lower scores. The most substantial estimated changes to depression scores occurred when replacing 30-min/day of MVPA with LIPA (+ 0.25 points), SB (+ 0.25 points), sleep (+ 0.22 points) and combination of remaining behaviours (+ 0.23 points).

Our findings indicate that relative time spent in MVPA, LIPA, SB and sleep are possible risk factors for depression outcomes in older adults which align with recent findings from several smaller studies [5, 6, 8, 12, 14]. There is consistent evidence that MVPA reduces incident depression and symptoms, a meta-analysis found that older adults who achieve sufficient weekly MVPA had a lower risk for depression (adjusted RR = 0.79, 95% CI: 0.73, 0.87). Contrary to our initial hypothesis MVPA was not strongly associated with reduced depression symptoms. This suggests that the response to MVPA may be blunted in later life and that increasing MVPA improves depression outcomes; however, permanence of these benefits is less certain [24]. Associations regarding LIPA are unclear, and evidence on whether it reduces symptoms and risk for depression is lacking [12]. Our results mirrored this inconsistency. The association between relative time spent in LIPA and depressive symptoms bordered statistical significance. Time substitutions were not statistically significant but suggested a reduction in symptoms when substituted with MVPA or sleep and increase in symptoms when replaced by SB. Our logistic regression models showed reduced risk for depression when replacing LIPA with MVPA and sleep, and increased risk when replaced with SB. While the effects of LIPA on depression outcomes in older adults remain unclear it may be a preferrable behaviour to SB. SB is not a clear determinant of depression with meta-analyses finding no evidence of an association with depression after adjusting for PA using traditional statistical methods (RR = 1.03, 95% CI: 0.90, 1.18) [5], and systematic reviews highlighting that the body of evidence is not indicative of a clear association in one direction, and that certain types of SB may lower risk of depression (e.g., socialising) while others increase it (e.g., television watching) [14]. We found SB was associated with increased risk of depression and symptoms, respectively; however, we did not account for the type of SB being performed. Future studies examining SB at the domain level may be useful for identifying which types are beneficial and detrimental. For sleep, a meta-analysis showed that an optimal sleep duration (7-8 h/night) is protective against depression, while short (< 6 h) and long (> 8 h) durations increased risk (RR = 1.27, 95% CI: 1.03, 1.56) [8]. Our results were aligned with the consensus in the literature that sleep is associated with lower symptoms and risk for depression in older adults and suggest adding time to sleep could improve depression outcomes so long as it does not replace PA.

Our analysis showed that associations held when using a CoDA framework to account for all behaviours in the daily movement behaviour composition.

We used compositional isotemporal substitution modelling to provide tangible estimates of how changes in time spent between movement behaviours affect depression symptoms scores. For instance, a recent meta-analysis found that high PA is associated with a 0.79 (95% CI: 0.72, 0.86) lower risk for depression compared to low PA [6]. Another meta-analysis found that high SB is associated with higher risk (RR = 1.10, 95% CI: 1.03, 1.19) for depression compared to low [5]. A meta-analysis examining LIPA, reported higher level was associated with lower risk for depression compared to low (RR = 0.77, 95% CI: 0.68, 0.86) [7]. Taken together, these results demonstrate the risks and benefits movement behaviours may have on depression symptoms, whereas our results demonstrate the importance of considering how time is allotted between behaviours. For instance, reducing daily SB by half an hour was associated with a larger effect size (OR = 0.85) when that time came from MVPA, and smaller effect size (OR = 0.94) for LIPA. These results are congruent with results from similar studies on adolescents [62] and adults [63]. The mechanisms through which increasing activity, reducing sedentary time and achieving optimal sleep duration modify depression symptoms in older adults are improved cardiovascular health and neuroplasticity, reduced inflammation, and increased feelings of self-efficacy and self-esteem [28].

Another advantage of compositional isotemporal modelling is that the asymmetry of effects for opposite time reallocations can be detected. This is a hallmark of the CoDA method that has important implications for health messaging and recommendations for the older adult population [64]. We found that removing MVPA from the geometric mean of MVPA was associated with greater increase in depression symptoms than the corresponding decrease in depression symptoms with adding MVPA to the geometric mean. This finding has been noted in other studies [26, 65] and suggests that encouraging older adults to preserve the time they spend being active, especially for those who do very little to begin with, may be more important than increasing it. This recommendation is particularly relevant, because older adults may not be physically able to increase their PA due to ageing related reduction in physical function.

Sex stratified analysis showed relative time spent in sleep was significantly associated in males, but not females; sleep time substitutions showed greater magnitude of effect in males. These findings aligned with Hoffman et al. (2022) who also used a CoDA framework and the same CES-D scale [26]. Our logistic regression models showed adding time to MVPA elicited lower odds for depression, and higher odds when adding time to LIPA and SB in females compared to males. These results suggest differences in depression symptomatology between older males and females. A number of explanations have been suggested for sex differences in depression symptoms, namely biological factors (e.g., hormonal differences), embodiment and learning of societal gender norms, coping mechanisms, and degree of reactivity [66]. For instance, women tend to experience more of the core emotional symptoms such as depressed mood and sadness, while men experience more somatic symptoms and anger. In our study, we found a greater protective effect of MVPA in females which we hypothesise may be attributable to the greater effect of MVPA on these emotional symptoms. Our initial hypothesis of a greater protective effect of sleep in females was not observed, and may stem from residual confounding from unmeasured disturbances in sleep quality (e.g., wake episodes, sleep latency, sleep efficiency), rather than sleep duration itself [67]. Older women may be more prone to sleep issues than men, such as insomnia and restlessness that influences their sleep quality, which has been shown to be a risk factor for depression symptoms on its own [17].

## Strengths and limitations

Several strategies were used to reduce risk of bias in this study. We used a large sample size of older adults from a nationally representative sample of Canadians which helps reduce bias from random variability. We calculated e-values to assess the risk of unmeasured confounding from the associations being reported and improve interpretability of results, which is recommended in observational studies [61]. Risk of reverse causality was reduced by modelling associations between baseline movement behaviour composition and depression symptoms at follow-up adjusting for symptoms at baseline, as well as sensitivity analysis that excluded all participants with clinical depression. We used CoDA to analyse associations between movement behaviours and depression symptoms, which allowed us to appropriately account for time spent in the rest of the day. CoDA also supports the estimation of the effects of time replacements between behaviours and provides a practical depiction of how changing time spent in one behaviour in relation to the remaining ones would occur. The a priori DAG allowed us to comprehensively determine which covariates need to be adjusted for in our models and reduce bias for effect estimates that can often be introduced with traditional adjustment methods (e.g., forward/backward elimination) in the form of under and over adjustment [68]. The breadth of variables available from CLSA, and sensitivity analyses helped us explore alternative hypotheses and improve our ability to estimate associations.

This study also has several limitations. It used self-reported PASE responses to estimate movement behaviour composition and a self-reported depression composite variable which are prone to recall and social desirability biases. PASE responses do not provide a full estimate of movement behaviours over a 24-h period. Therefore, it is important to recognise that there is likely non differential misclassification that may have biased our results. PASE is likely to underestimate both PA and SB because the range of included activities are not exhaustive. Regarding sleep, participants responded to a single question about the average amount of time they spent sleeping on a given night within the last month. This is a crude measure of daily sleep duration that may have overestimated sleep in the overall composition. These are common issues for researchers using CoDA on self-reported movement behaviour data. Regardless, the CoDA approach is preferred because these data still carry relative information constrained to a finite period that needs to be respected and the relative structure derived from the ratios between behaviours remains of key importance [69].

It is important to note that although objective measures are generally considered superior to self-reported measures, both have advantages and disadvantages that may modify associations between activity and depression symptoms [70]. Self-reported measures provide a better assessment of contextual specific information about the behaviour [active SB (e.g., socialising) versus passive SB (e.g., watching television)] and can capture activity undetectable to accelerometry (e.g., bicycling). Conversely, objective measures provide better assessment of movement behaviour patterns and time accumulation. As well, algorithms used in device-based measures may alter validity of exposure assessment [70]. There is also inconsistent evidence if a seven-day measurement of activity is indicative of a typical week for older adults [71, 72]. For self-reported measures older adults may struggle to recall the activity they did or inflate the amount of activity they report while those fitted with an accelerometer may increase their activity duration during the study period.

A large proportion of participants (31.5%) were removed from our final study sample due to insufficient PASE data. If the relationship between movement behaviour composition and depression symptoms was different between those with and without adequate PASE data, our results may be biased. In our study sample mean daily time in MVPA was 55 min compared to 20 min per day in the 2018 Canadian Health Measures Survey for older adults ≥ 65 years of age. While it is difficult to make direct comparisons, this difference shows that estimates of time spent in movement behaviours from our sample may not be entirely representative of the older adult population [73] and it may be more ‘active’ than the average older adult population.

While the full comprehensive cohort is comparable to the general population across several sociodemographic and health factors and estimates from other nationally representative surveys with high response rates [e.g., Canadian Community Health Survey-Health Aging (CCHS-HA)] its representativeness is limited due to self-selection, as those who agreed to participate were healthier, wealthier, white and more educated; as well, strictly older adults were included; therefore, our results are only generalisable to this demographic [32]. Issues with representativeness are important to note because they can lead to collider bias whereby two variables (e.g., exposure and outcome) cause a third variable (e.g., participation in the study), with the effect of the third variable distorting the causal association being measured [74]. Still, risk of this bias in our study was increased as movement behaviour (exposure) and depression (outcome) estimates in the CLSA were inconsistent with other nationally representative samples, which may have affected participation [75]. In our study sample, 10.9% of participants had clinical depression diagnosis, while it is approximately 7% in the CCHS-HA [75]; however, more recent estimates suggest that prevalence is 10% [76].

The e-values indicate low risk of unmeasured confounding nullifying our main findings. One exception was for the effects of substituting time out of MVPA into sleep. Sleep quality is an important factor for depression symptoms that may have confounded estimates of replacing MVPA, LIPA and SB with sleep, and may explain why we did not see a significant association for the sleep base model in females. We only included measures of sleep duration, but sleep quality (e.g., sleep efficiency, wake-rest cycle) and timing of sleep are also important considerations for depression and mental health [77]. Assessing sleep quality usually requires rigorous measures that are impractical in large-scale studies, such as direct observation and polysomnography.

Overall, it remains possible that several unmeasured confounding variables may accumulate and have this effect on our main findings. We took steps in our analysis to reduce risk of reverse causation; however, there may still be bias attributable to measurement error of baseline depression symptoms. Baseline depression symptoms were measured with the same composite score, limiting us from estimating incidence of possible cases at baseline; however, we were able to do this in our logistic regression models.

## Implications and future directions

Our findings suggest daily movement behaviour composition is associated with depression outcomes in older adults. Preserving and increasing MVPA showed to be the most important behaviour; limiting SB and achieving optimal sleep duration also reduced depression symptoms. Interventions aimed at promoting a healthy movement behaviour profile should consider how reallocating time between different behaviours affect depression symptoms. For example, we estimated that replacing MVPA with any other behaviour was associated with the highest depression symptoms scores; however, the greatest effect was seen when that time came from SB. Our results suggest that even small changes of less than an hour (e.g., 30-min) could change depression symptoms, and that maintaining MVPA throughout the later years is important. For example, reducing SB by 30-min and replacing it with brisk walking (MVPA) may be adequate for reducing depression symptoms, and a realistic goal. Reducing SB and increasing time spent in the remaining healthier behaviours may have a smaller effect on reducing depression symptoms than MVPA alone, but is more reasonable and sustainable long-term, as light activity and ensuring optimal sleep time are easier to achieve, typically more pleasurable, and require less motivation to maintain than intense activity [78].

Studies investigating effects of movement behaviours on individual symptoms of depression (e.g., low mood) can elucidate roles of specific behaviours. Use of objective measures for all parts of the movement behaviour composition can be helpful for validating the current study findings. This is especially important for sleep because it is often gathered through self-reported measures even in CoDA studies using accelerometry [20, 58] owing to detection issues with wrist- and waist-worn activity devices, such as measuring onset of biological sleep and distinguishing between SB and sleep [79].

Future studies can aim to incorporate both subjective and objective measures where possible to provide more contextual information about the activities being performed, or timing of the activity within the day and their effects on depression, thereby reducing potential misclassification. Implementation of time-use recalls may be a promising alternative that addresses the shortcomings of self-reported and device gathered movement behaviour data, by providing good validity and resolution and context specific information [80].

There is evidence that passive sedentary behaviours are associated with greater depression symptoms than active behaviours [5]. Choosing how to spend sedentary time can be a useful strategy for lowering depression risk for those who are not active or unable to do activity. For example, replacing 30-min of television with 30-min of reading may mitigate or improve depression outcomes. Researchers can also consider collecting repeated measures of the movement behaviour composition to better measure habitual movement patterns.

The current study findings also demonstrate the distinction of understanding associations between movement behaviours and depression in a daily context. More studies using a CoDA framework to appropriately account for daily time in all behaviours and estimate replacement effects instead of analysing time spent in one behaviour on its own without appropriate adjustments, are necessary to advance research in the movement behaviour epidemiology field focussed on older adult mental health [12–14, 23, 77]. Future studies can also consider prospective design to establish temporality, and interventional studies to validate findings from those studies.

## Conclusions

This study found that movement behaviour composition is associated with depression outcomes in older adults. Further consideration of how to optimally distribute time spent in each behaviour over the course of the day is warranted. Our estimates showed replacing time spent in MVPA with time from LIPA, SB or sleep can increase depression symptoms with replacements as small as 30-min. Therefore, maintaining time spent in MVPA may be an important consideration for mitigating depression throughout the later years. Ultimately, a comprehensive 24-h lifestyle management approach will be beneficial for effectively reducing depression symptoms and promoting healthy ageing.

To advance the field, a greater adoption of compositional methods is required. Greater emphasis on understanding the relative effects of time spent in LIPA on depression outcomes is needed, as well as addressing different types of SB, and controlling for sleep quality.

## Supplementary Information

**Additional file 1:**
**Figure S1.** Results of power analysis; **Figure S2.** DAG of our causal assumptions; **Figure S3. **Outcome distribution and additional details; **Table S1.** Compositional (geometric) and arithmetic means of time spent in movement behaviours for sex stratifications; **Table S2.** compositional variation matrices of time spent in movement behaviours for sex stratifications; **Table S3.** Base model results for sex stratifications; **Tables S4-S7; Results S1. **Sensitivity analysis results.

**Additional file 2:**
**Tables S1-S5**. Time reallocation (15–120 min) estimates for full sample and sex stratifications; **Tables S6-S11.** Odds ratios for depression for full sample and sex stratifications; **Figures S1-S4.** Graphical representations of time estimates for each movement behaviour for sex stratifications.

**Additional file 3.** STROBE checklist.

## Data Availability

Data are available from the Canadian Longitudinal Study on Aging (www.clsa-elcv.ca) for researchers who meet the criteria for access to de-identified CLSA data.

## Abbreviations

BMI: Body mass index
CCHS-HA: Canadian Community Health Survey-Healthy Aging
CES-D: Center for Epidemiologic Studies Depression Scale
CLSA: Canadian Longitudinal Study on Aging
CI: Confidence interval
CoDA: Compositional data analysis
DAG: Directed acyclic graph
ilr: Isometric log-ratio
LIPA: Light-intensity physical activity
MET: Metabolic equivalent of task
MVPA: Moderate-to-vigorous physical activity
OR: Odds ratio
PA: Physical activity
PASE: Physical Activity Scale for the Elderly
PURE: Prospective Urban Rural Epidemiological diet quality score
RR: Relative risk
SB: Sedentary behaviour
SD: Standard deviation
STROBE: Strengthening the Reporting of Observational Studies in Epidemiology

## Declarations

### Disclaimer

The opinions expressed in this manuscript are the author's own and do not reflect tdian Longitudinal Study on Aging.
