# Supplementary material for: Impacts of physical activity, sedentary behaviour, and sleep on depression symptoms in Canadian older adults 65 years of age and above: a compositional data analysis of the Canadian Longitudinal Study on Aging
Source: J Act Sedentary Sleep Behav. 2024 Mar 8;3:8. doi: 10.1186/s44167-024-00047-7 (PMC11960353; doi:10.1186/s44167-024-00047-7)
Supplement: Supplementary file 1 — Additional file 1: Figure S1. Results of power analysis; Figure S2. DAG of our causal assumptions; Figure S3. Outcome distribution and additional details; Table S1. Compositional (geometric) and arithmetic means of time spent in movement behaviours for sex stratifications; Table S2. compositional variation matrices of time spent in movement behaviours for sex stratifications; Table S3. Base model results for sex stratifications; Tables S4-S7; Results S1. Sensitivity analysis results. [file 44167_2024_47_MOESM1_ESM.docx]

**Supplementary Materials-File 1**

**Figure 1**. Power analysis result for medium effect size (f^2^ = 0.15).


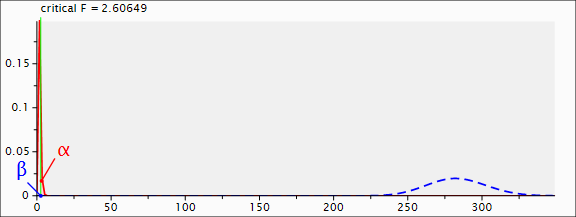


Power calculations completed using G*Power to estimate power of the study sample to detect small (f^2^ = 0.02), medium [shown above, (f^2^ = 0.15)] and large effect sizes (f^2^ = 0.35) with α set to 0.05 (1,2).

**Figure 2**. Directed acyclic graph (DAG) depicting assumptions underlying causal associations between covariates used in this analysis.


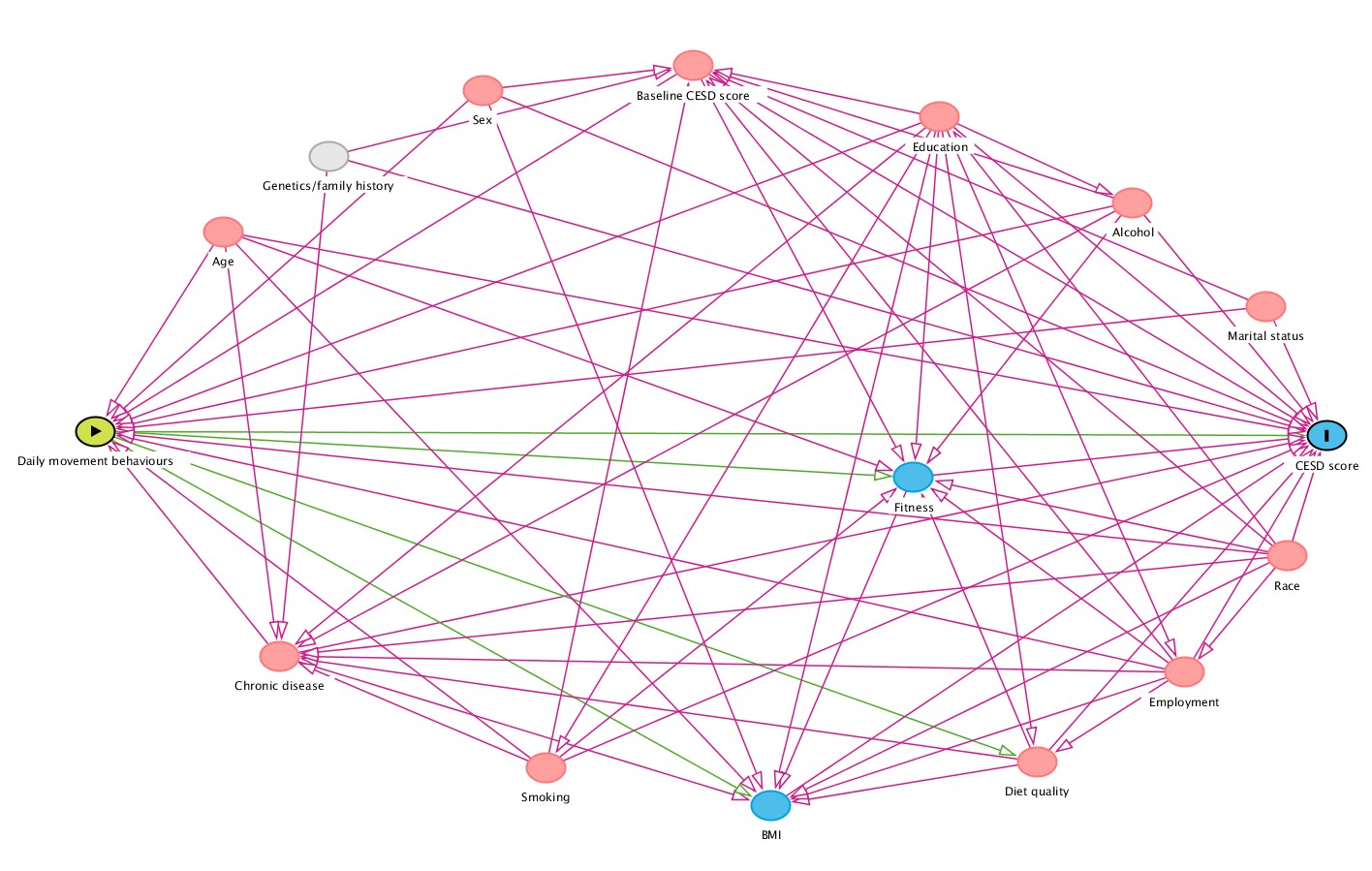


Dagitty online software package used to create DAG and legend ([www.dagitty.net](http://www.dagitty.net)) (3).

BMI = body mass index, CES-D = Centre for Epidemiological Studies Depression Scale

Legend:


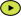
 exposure


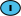
 outcome


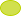
 ancestor of exposure


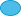
 ancestor of outcome


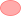
 ancestor of exposure and outcome


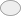
 unobserved (latent)


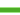
 causal path


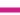
 biasing path

**Figure 3**. CES-D 10 score distribution


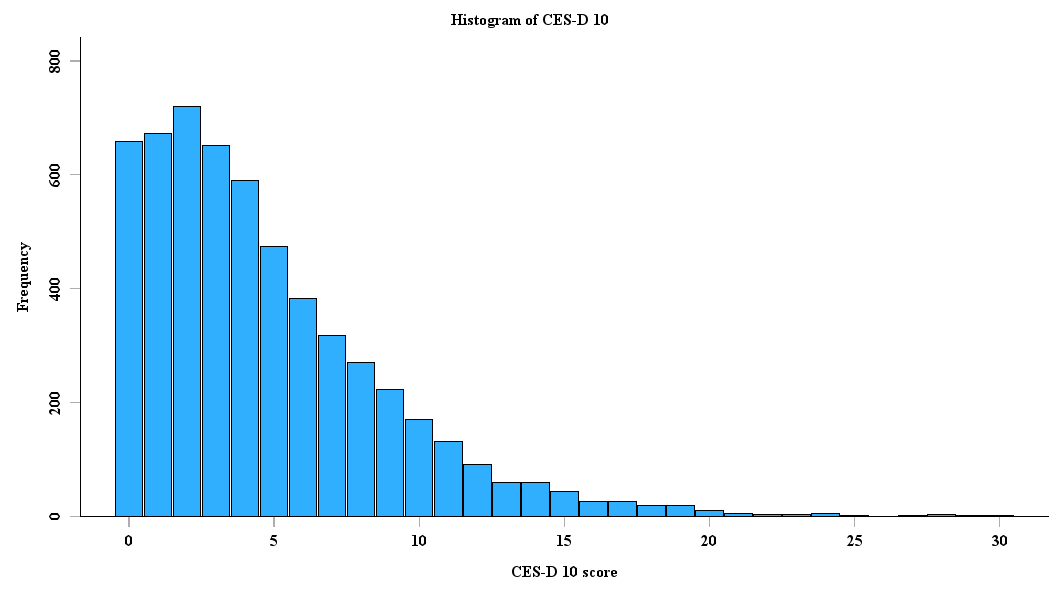


Residual deviance: 13,749 on 5,618 degrees of freedom indicating overdispersion; the dispersion parameter was 2.45, and variance (17) was greater than mean outcome (4.7).

**Table 1**. Compositional (geometric) and arithmetic means of time spent in moderate-to-vigorous physical activity, light-intensity physical activity, sedentary behaviour and sleep per day for full sample, and sex stratifications.

|  | Compositional | | Arithmetic | |  |
| --- | --- | --- | --- | --- | --- |
| Movement behaviour | Mean  (hours:minutes) | Proportion | Mean  (hours:minutes ±SD) | Proportion | |
| **MVPA** |  |  |  |  | |
| Full sample | 0:36 | 0.04 | 0:55 (±1:01) | 0.06 | |
| Males | 0:42 | 0.05 | 1:04 (±1:10) | 0.07 | |
| Females | 0:31 | 0.04 | 0:46 (±0:49) | 0.05 | |
| **LIPA** |  |  |  |  | |
| Full sample | 2:49 | 0.20 | 3:22 (±2:13) | 0.22 | |
| Males | 3:03 | 0.21 | 3:36 (±2:21) | 0.23 | |
| Females | 2:36 | 0.19 | 3:06 (±2:02) | 0.21 | |
| **SB** |  |  |  |  | |
| Full sample | 3:54 | 0.28 | 4:17 (±1:38) | 0.28 | |
| Males | 3:56 | 0.27 | 4:20 (±1:42) | 0.27 | |
| Females | 3:52 | 0.28 | 4:15 (±1:34) | 0.28 | |
| **Sleep** |  |  |  |  | |
| Full sample | 6:47 | 0.48 | 6:54 (±1:11) | 0.44 | |
| Males | 6:49 | 0.47 | 6:56 (±1:08) | 0.43 | |
| Females | 6:45 | 0.49 | 6:52 (±1:14) | 0.46 | |

SD, standard deviation

**Table 2**. Compositional variation matrices of time spent in MVPA, LIPA, SB, and sleep for full sample, and sex stratifications.

| **Sample** | MVPA | LIPA | SB | Sleep |
| --- | --- | --- | --- | --- |
| **Full** |  |  |  |  |
| MVPA | 0 | 0.43 | 0.45 | 0.65 |
| LIPA | 0.43 | 0 | 0.99 | 0.93 |
| SB | 0.45 | 0.99 | 0 | 0.90 |
| Sleep | 0.65 | 0.93 | 0.90 | 0 |
| **Males** |  |  |  |  |
| MVPA | 0 | 0.52 | 0.56 | 0.75 |
| LIPA | 0.52 | 0 | 0.99 | 0.85 |
| SB | 0.56 | 0.99 | 0 | 0.95 |
| Sleep | 0.75 | 0.85 | 0.95 | 0 |
| **Females** |  |  |  |  |
| MVPA | 0 | 0.30 | 0.30 | 0.50 |
| LIPA | 0.30 | 0 | 0.99 | 0.99 |
| SB | 0.30 | 0.99 | 0 | 0.94 |
| Sleep | 0.50 | 0.99 | 0.94 | 0 |

**Table 3**. Compositional negative binomial regression model estimates for depression symptoms for full sample, and sex stratifications.

|  | **Model** | **MVPA** |  | **LIPA** |  | **SB** |  | | **Sleep** |  | |
| --- | --- | --- | --- | --- | --- | --- | --- | --- | --- | --- | --- |
|  | ***p*-value** | **exp(**$\boldsymbol{\gamma}$**)* (95%CI)** | ***p*-value** | **exp(**$\boldsymbol{\gamma}$**)* (95%CI)** | ***p*-value** | **exp(**$\boldsymbol{\gamma}$**)*(95%CI)** | | ***p*-value** | **exp(**$\boldsymbol{\gamma}$**)* (95%CI)** | | ***p*-value** |
| **Unadjusted** |  |  |  |  |  |  | |  |  | |  |
| Full | < 0.001 | 0.92 (0.90, 0.95) | < 0.001 | 0.98 (0.93, 1.02) | 0.32 | 1.27 (1.21, 1.34) | | < 0.001 | 0.80 (0.72, 0.90) | | < 0.001 |
| Males | < 0.001 | 0.95 (0.91, 0.99) | 0.01 | 1.03 (0.96, 1.10) | 0.45 | 1.28 (1.19, 1.38) | | < 0.001 | 0.80 (0.72, 0.90) | | < 0.001 |
| Females | < 0.001 | 0.94 (0.90, 0.98) | 0.006 | 0.97 (0.91, 1.03) | 0.32 | 1.29 (1.20, 1.38) | | < 0.001 | 0.85 (0.77, 0.95) | | 0.003 |
| **Adjusted** |  |  |  |  |  |  | |  |  | |  |
| Full | < 0.001 | 0.97 (0.94, 0.99) | 0.02 | 1.04 (0.99, 1.08) | 0.08 | 1.09 (1.04, 1.15) | | < 0.001 | 0.91 (0.85, 0.97) | | 0.005 |
| Males | < 0.001 | 0.97 (0.93, 1.00) | 0.08 | 1.05 (0.99, 1.12) | 0.10 | 1.10 (1.03, 1.18) | | 0.006 | 0.89 (0.81, 0.98) | | 0.02 |
| Females | < 0.001 | 0.96 (0.93, 1.00) | 0.06 | 1.03 (0.97, 1.08) | 0.40 | 1.07 (1.01, 1.15) | | 0.03 | 0.94 (0.86, 1.03) | | 0.19 |

*Note*: Adjusted for age, sex, race, employment status, education level, marital status, smoking status, alcohol consumption, diet quality score, comorbidity burden score, baseline depression symptoms. *Exponential of regression coefficient ($\gamma$). These values represent the proportional unit change in depression symptoms score per unit increase in the associated *ilr* coordinate, as time allocated to the 24h movement behaviour in the numerator against the geometric mean of the others in the denominator. In these models, only *z*_1_ is interpretable as it contains the relative information for all 24h movement behaviours (i.e., MVPA, LIPA, sedentary behaviour and sleep). The p-value indicates a statistically significant association between the 24-h movement behaviour and depression symptoms score after accounting for the time spent in the remaining movement behaviour.

**Sensitivity analysis results**

**Table 4:** Estimated change in depression symptoms as measured by CES-D score scale that would occur by displacing 30-minutes between movement behaviours *after excluding people with depression.*

| **Remove 30-min**  **per day**  **from** |  | **Add 30-min per day to** | | |
| --- | --- | --- | --- | --- |
|  | **MVPA** | **LIPA** | **SB** | **Sleep** |
| MVPA | – | 0.19 (0.09, 0.28) | 0.19 (0.10, 0.29) | 0.16 (0.07, 0.26) |
| LIPA | -0.01 (-0.10, 0.09) | – | 0.01 (-0.09, 0.10) | -0.01 (-0.11, 0.08) |
| SB | -0.04 (-0.13, 0.06) | -0.01 (-0.10, 0.08) | – | -0.02 (-0.12, 0.07) |
| Sleep | 0.01 (-0.08, 0.11) | 0.02 (-0.08, 0.11) | 0.02 (-0.07, 0.12) | – |

*Note*: Data presented as estimated points change in CES-D score (95% confidence interval). All estimates adjusted for age, sex, race, employment status, education level, marital status, smoking status, alcohol consumption, diet quality score, comorbidity burden score, baseline depression symptoms. Values reflect estimated points change in CES-D score with re-allocating 30-minutes from the movement behaviour in the column to the movement behaviour in the row using the mean movement behaviour composition as the reference. For example, replacing 30-minutes of MVPA with 30-minutes of LIPA would result in an increase of 0.19 points in depression symptoms. Lower CES-D score indicates less depression symptoms, higher score indicates more depression symptoms. MVPA moderate-to-vigorous physical activity, LIPA light-intensity physical activity, SB sedentary behaviour.

**Table 5**: Estimated change in depression symptoms as measured by CES-D score scale that would occur by displacing 30-minutes between movement behaviours after excluding people with depression *after removing 10% of study sample by random selection.*

| **Remove 30-min**  **per day**  **from** |  | **Add 30-min per day to** | | |
| --- | --- | --- | --- | --- |
|  | **MVPA** | **LIPA** | **SB** | **Sleep** |
| MVPA | – | 0.29 (0.19, 0.39) | 0.29 (0.19, 0.39) | 0.26 (0.16, 0.36) |
| LIPA | -0.03 (-0.12, 0.07) | – | 0.01 (-0.10, 0.10) | -0.02 (-0.11, 0.08) |
| SB | -0.04 (-0.14, 0.06) | -0.01 (-0.11, 0.09) | – | -0.02 (-0.12, 0.08) |
| Sleep | 0.01 (-0.09, 0.10) | 0.02 (-0.08, 0.12) | 0.02 (-0.08, 0.12) | – |

*Note*: Data presented as estimated points change in CES-D score (95% confidence interval). All estimates adjusted for age, sex, race, employment status, education level, marital status, smoking status, alcohol consumption, diet quality score, comorbidity burden score, baseline depression symptoms. Values reflect estimated points change in CES-D score with re-allocating 30-minutes from the movement behaviour in the column to the movement behaviour in the row using the mean movement behaviour composition as the reference. For example, replacing 30-minutes of MVPA with 30-minutes of LIPA would result in an increase of 0.29 points in depression symptoms. Lower CES-D score indicates less depression symptoms, higher score indicates more depression symptoms. MVPA moderate-to-vigorous physical activity, LIPA light-intensity physical activity, SB sedentary behaviour.

**Table 6**. Estimated change in depression symptoms as measured by CES-D score scale that would occur by displacing 30-minutes from one movement behaviour to remaining behaviours proportionally *after excluding people with depression.*

| **Movement behaviour** | **Remove 30-min of column**  **behaviour and add to remaining behaviours** | **Add 30-min to column behaviour from remaining two behaviours** |
| --- | --- | --- |
| MVPA | 0.18 (0.08, 0.27) | -0.02 (-0.12, 0.07) |
| LIPA | -0.01 (-0.10, 0.09) | 0.01 (-0.09, 0.10) |
| SB | -0.02 (-0.11, 0.07) | 0.02 (-0.07, 0.11) |
| Sleep | 0.02 (-0.07, 0.12) | -0.02 (-0.12, 0.07) |

*Note*: Data presented as estimated points change in CES-D score (95% confidence interval). All estimates adjusted for age, sex, race, employment status, education level, marital status, smoking status, alcohol consumption, diet quality score, comorbidity burden score, baseline depression symptoms. Values reflect estimated points change in CES-D score with re-allocating time from one behaviour to the remaining movement behaviours proportionally (or vice versa) using the mean movement behaviour composition as the reference. For example, replacing 30-minutes of MVPA with 7.69-minutes LIPA, 10.56-minutes SB and 18.25-minutes sleep would result in an increase of 0.18 points in depression symptoms. Lower CES-D score indicates less depression symptoms, higher score indicates more depression symptoms. MVPA moderate-to-vigorous physical activity, LIPA light-intensity physical activity, SB sedentary behaviour.

**Table 7**. Estimated change in depression symptoms as measured by CES-D score scale that would occur by displacing 30-minutes from one movement behaviour to remaining behaviours proportionally *after removing 10% of study sample by random selection.*

| **Movement behaviour** | **Remove 30-min of column**  **behaviour and add to remaining behaviours** | **Add 30-min to column behaviour from remaining two behaviours** |
| --- | --- | --- |
| MVPA | 0.28 (0.18, 0.38) | -0.03 (-0.13, 0.07) |
| LIPA | -0.01 (-0.11, 0.09) | 0.01 (-0.09, 0.11) |
| SB | -0.02 (-0.12, 0.08) | 0.02 (-0.08, 0.12) |
| Sleep | 0.02 (-0.08, 0.12) | -0.02 (-0.12, 0.08) |

*Note*: Data presented as estimated points change in CES-D score (95% confidence interval). All estimates adjusted for age, sex, race, employment status, education level, marital status, smoking status, alcohol consumption, diet quality score, comorbidity burden score, baseline depression symptoms. Values reflect estimated points change in CES-D score with re-allocating time from one behaviour to the remaining movement behaviours proportionally (or vice versa) using the mean movement behaviour composition as the reference. For example, replacing 30-minutes of MVPA with 7.52-minutes LIPA, 10.50-minutes SB and 17.98-minutes sleep would result in an increase of 0.28 points in depression symptoms. Lower CES-D score indicates less depression symptoms, higher score indicates more depression symptoms. MVPA moderate-to-vigorous physical activity, LIPA light-intensity physical activity, SB sedentary behaviour.

**Results 1**: E-values

For depression scores, the e-values as incident rate ratios for substituting 30 minutes of MVPA with LIPA, SB, and sleep were 1.47 (CI = 1.16), 2.22 (CI = 1.88) and 0.41 (CI = 1), respectively.

**References**

1. Cohen J. Statistical power analysis. Curr Dir Psychol Sci. 1992;1(3):98–101.

2. Faul F, Erdfelder E, Lang A-G, Buchner A. G*Power 3: A flexible statistical power analysis program for the social, behavioral, and biomedical sciences. Behav Res Methods. 2007;39(2):175–91.

3. Textor J, van der Zander B, Gilthorpe MS, Liśkiewicz M, Ellison GTH. Robust causal inference using directed acyclic graphs: the R package ‘dagitty.’ Int J Epidemiol. 2016;45(6):1887–94.
