# Supplementary material for: Impacts of physical activity, sedentary behaviour, and sleep on depression symptoms in Canadian older adults 65 years of age and above: a compositional data analysis of the Canadian Longitudinal Study on Aging
Source: J Act Sedentary Sleep Behav. 2024 Mar 8;3:8. doi: 10.1186/s44167-024-00047-7 (PMC11960353; doi:10.1186/s44167-024-00047-7)
Supplement: Supplementary file 2 — Additional file 2: Tables S1-S5. Time reallocation (15–120 min) estimates for full sample and sex stratifications; Tables S6-S11. Odds ratios for depression for full sample and sex stratifications; Figures S1-S4. Graphical representations of time estimates for each movement behaviour for sex stratifications. [file 44167_2024_47_MOESM2_ESM.docx]

| **FULL SAMPLE (n = 5,643)** | | | | | | | | | | | | |
| --- | --- | --- | --- | --- | --- | --- | --- | --- | --- | --- | --- | --- |
| Remove time from: | Add time to MVPA (minutes) | | | | |  | Add time to: | Remove time from MVPA (minutes) | | | | |
|  | 15 | 30 | 45 | 60 | 75 |  |  | 15 | 30 | 45 | 60 | 75 |
| LIPA | -0.01 (-0.10, 0.09) | -0.02 (-0.11, 0.08) | -0.02 (-0.11, 0.07) | -0.03 (-0.12, 0.07) | -0.03 (-0.13, 0.06) |  | LIPA | 0.02 (-0.07, 0.12) | 0.25 (0.15, 0.34) |  |  |  |
| SB | -0.02 (-0.12, 0.07) | -0.04 (-0.14, 0.05) | -0.06 (-0.15, 0.04) | -0.07 (-0.16, 0.02) | -0.09 (-0.18, 0.00) |  | SB | 0.02 (-0.07, 0.12) | 0.25 (0.15, 0.34) |  |  |  |
| Sleep | 0.01 (-0.09, 0.10) | 0.01 (-0.09, 0.10) | 0.01 (-0.08, 0.11) | 0.02 (-0.08, 0.11) | 0.02 (-0.07, 0.11) |  | Sleep | 0.01 (-0.08, 0.11) | 0.22 (0.13, 0.32) |  |  |  |
|  | | | | | | | | | | | | |
| **MALES (n = 2,931)** | | | | | | | | | | | | |
| Remove time from: | Add time to MVPA (minutes) | | | | |  | Add time to: | Remove time from MVPA (minutes) | | | | |
|  | 15 | 30 | 45 | 60 | 75 |  |  | 15 | 30 | 45 | 60 | 75 |
| LIPA | -0.01 (-0.14, 0.12) | -0.02 (-0.15, 0.11) | -0.03 (-0.15, 0.10) | -0.03 (-0.16, 0.10) | -0.04 (-0.17, 0.09) |  | LIPA | 0.02 (-0.11, 0.15) | 0.27 (0.14, 0.40) |  |  |  |
| SB | -0.02 (-0.15, 0.11) | -0.04 (-0.17, 0.09) | -0.05 (-0.18, 0.07) | -0.07 (-0.20, 0.06) | -0.08 (-0.21, 0.05) |  | SB | 0.02 (-0.11, 0.15) | 0.27 (0.14, 0.40) |  |  |  |
| Sleep | 0.01 (-0.12, 0.14) | 0.01 (-0.12, 0.15) | 0.01 (-0.11, 0.15) | 0.03 (-0.10, 0.16) | 0.04 (-0.10, 0.16) |  | Sleep | 0.01 (-0.12, 0.14) | 0.24 (0.11, 0.37) |  |  |  |
|  | | | | | | | | | | | | |
| **FEMALES (n = 2,712)** | | | | | | | | | | | | |
| Remove time from: | Add time to MVPA (minutes) | | | | |  | Add time to: | Remove time from MVPA (minutes) | | | | |
|  | 15 | 30 | 45 | 60 | 75 |  |  | 15 | 30 | 45 | 60 | 75 |
| LIPA | -0.01 (-0.15, 0.13) | -0.02 (-0.16, 0.12) | -0.03 (-0.17, 0.11) | -0.03 (-0.17, 0.11) | -0.04 (-0.18, 0.10) |  | LIPA | 0.03 (-0.11, 0.17) | 0.26 (0.12, 0.40) |  |  |  |
| SB | -0.03 (-0.17, 0.11) | -0.05 (-0.19, 0.09) | -0.06 (-0.20, 0.08) | -0.08 (-0.22, 0.06) | -0.10 (-0.24, 0.04) |  | SB | 0.03 (-0.11, 0.17) | 0.26 (0.12, 0.40) |  |  |  |
| Sleep | -0.01 (-0.15, 0.14) | -0.01 (-0.15, 0.13) | -0.01 (-0.15, 0.13) | -0.01 (-0.15, 0.13) | -0.01 (-0.15, 0.13) |  | Sleep | 0.02 (-0.12, 0.16) | 0.25 (0.11, 0.39) |  |  |  |

**Supplementary Table 1.** Estimates for depression symptoms as measured by CES-D score scale when displacing time into or out of MVPA.

*Note*: Data presented as estimated points change in CES-D score (95% confidence interval). All estimates adjusted for age, sex, race, employment status, education level, marital status, smoking status, alcohol consumption, diet quality score, comorbidity burden score, baseline depression symptoms. Values reflect estimated points change in CES-D score with re-allocating time from the movement behaviour in the column to the movement behaviour in the row using the mean movement behaviour composition as the reference. For example, replacing 30-minutes of MVPA with 30-minutes of LIPA would result in an increase of 0.25 points in depression symptoms in the full sample. Lower CES-D score indicates less depression symptoms, higher score indicates more depression symptoms. MVPA moderate-to-vigorous physical activity, LIPA light-intensity physical activity, SB sedentary behaviour.

| **FULL SAMPLE (n = 5,643)** | | | | | | | | | | | | |
| --- | --- | --- | --- | --- | --- | --- | --- | --- | --- | --- | --- | --- |
| Remove time from: | Add time to LIPA (minutes) | | | | |  | Add time to: | Remove time from LIPA (minutes) | | | | |
|  | 15 | 30 | 60 | 90 | 120 |  |  | 15 | 30 | 60 | 90 | 120 |
| MVPA | 0.02 (-0.07, 0.12) | 0.25 (0.15, 0.34) |  |  |  |  | MVPA | -0.01 (-0.10, 0.09) | -0.02 (-0.11, 0.08) | -0.03 (-0.12, 0.07) |  |  |
| SB | -0.01 (-0.10, 0.09) | -0.01 (-0.10, 0.08) | -0.02 (-0.11, 0.07) | -0.03 (-0.13, 0.06) | -0.05 (-0.15, 0.04) |  | SB | 0.01 (-0.09, 0.10) | 0.01 (-0.09, 0.10) | 0.01 (-0.09, 0.10) | 0.01 (-0.09, 0.10) | -0.01 (-0.10, 0.09) |
| Sleep | 0.01 (-0.09, 0.10) | 0.02 (-0.08, 0.11) | 0.03 (-0.06, 0.13) | 0.05 (-0.05, 0.14) | 0.06 (-0.03, 0.16) |  | Sleep | -0.01 (-0.10, 0.09) | -0.01 (-0.11, 0.08) | -0.03 (-0.12, 0.07) | -0.05 (-0.14, 0.05) |  |
|  | | | | | | | | | | | | |
| **MALES (n = 2,931)** | | | | | | | | | | | | |
| Remove time from: | Add time to LIPA (minutes) | | | | |  | Add time to: | Remove time from LIPA (minutes) | | | | |
|  | 15 | 30 | 60 | 90 | 120 |  |  | 15 | 30 | 60 | 90 | 120 |
| MVPA | 0.02 (-0.11, 0.15) | 0.27 (0.14, 0.40) |  |  |  |  | MVPA | -0.01 (-0.14, 0.12) | -0.02 (-0.15, 0.11) | -0.03 (-0.16, 0.10) |  |  |
| SB | -0.01 (-0.13, 0.13) | -0.01 (-0.14, 0.12) | -0.02 (-0.15, 0.11) | -0.03 (-0.16, 0.10) | -0.05 (-0.18, 0.08) |  | SB | 0.01 (-0.13, 0.13) | 0.01 (-0.13, 0.13) | 0.01 (-0.13, 0.13) | 0.01 (-0.13, 0.13) | -0.01 (-0.14, 0.12) |
| Sleep | 0.01 (-0.12, 0.14) | 0.02 (-0.11, 0.15) | 0.04 (-0.09, 0.17) | 0.06 (-0.07, 0.19) | 0.08 (-0.05, 0.21) |  | Sleep | -0.01 (-0.14, 0.12) | -0.02 (-0.15, 0.11) | -0.04 (-0.17, 0.09) | -0.06 (-0.19, 0.07) |  |
|  | | | | | | | | | | | | |
| **FEMALES (n = 2,712)** | | | | | | | | | | | | |
| Remove time from: | Add time to LIPA (minutes) | | | | |  | Add time to: | Remove time from LIPA (minutes) | | | | |
|  | 15 | 30 | 60 | 90 | 120 |  |  | 15 | 30 | 60 | 90 | 120 |
| MVPA | 0.03 (-0.11, 0.17) | 0.26 (0.12, 0.40) |  |  |  |  | MVPA | -0.01 (-0.15, 0.13) | -0.02 (-0.16, 0.12) | -0.03 (-0.17, 0.11) |  |  |
| SB | -0.01 (-0.14, 0.14) | -0.01 (-0.15, 0.13) | -0.02 (-0.16, 0.12) | -0.03 (-0.17, 0.11) | -0.05 (-0.19, 0.09) |  | SB | 0.01 (-0.14, 0.14) | 0.01 (-0.14, 0.14) | 0.01 (-0.15, 0.13) | 0.01 (-0.15, 0.13) | -0.01 (-0.14, 0.14) |
| Sleep | 0.01 (-0.14, 0.15) | 0.01 (-0.13, 0.15) | 0.02 (-0.12, 0.16) | 0.03 (-0.11, 0.17) | 0.04 (-0.10, 0.18) |  | Sleep | -0.01 (-0.14, 0.14) | -0.01 (-0.15, 0.13) | -0.02 (-0.16, 0.12) | 0.03 (-0.17, 0.11) |  |

**Supplementary Table 2.** Estimates for depression symptoms as measured by CES-D score scale when displacing time into or out of LIPA.

*Note*: Data presented as estimated points change in CES-D score (95% confidence interval). All estimates adjusted for age, sex, race, employment status, education level, marital status, smoking status, alcohol consumption, diet quality score, comorbidity burden score, baseline depression symptoms. Values reflect estimated points change in CES-D score with re-allocating time from the movement behaviour in the column to the movement behaviour in the row using the mean movement behaviour composition as the reference. For example, replacing 30-minutes of LIPA with 30-minutes of MVPA would result in a decrease of 0.02 points in depression symptoms in the full sample. Lower CES-D score indicates less depression symptoms, higher score indicates more depression symptoms. MVPA moderate-to-vigorous physical activity, LIPA light-intensity physical activity, SB sedentary behaviour.

**Supplementary Table 3.** Estimates for depression symptoms as measured by CES-D score scale when displacing time into or out of SB.

| **FULL SAMPLE (n = 5,643)** | | | | | | | | | | | | |
| --- | --- | --- | --- | --- | --- | --- | --- | --- | --- | --- | --- | --- |
| Remove time from: | Add time to SB (minutes) | | | | |  | Add time to: | Remove time from SB (minutes) | | | | |
|  | 15 | 30 | 60 | 90 | 120 |  |  | 15 | 30 | 60 | 90 | 120 |
| MVPA | 0.02 (-0.07, 0.12) | 0.25 (0.15, 0.34) |  |  |  |  | MVPA | -0.02 (-0.12, 0.07) | -0.04 (-0.14, 0.05) | -0.07 (-0.16, 0.02) |  |  |
| LIPA | 0.01 (-0.09, 0.10) | 0.01 (-0.09, 0.10) | 0.01 (-0.09, 0.10) | 0.01 (-0.09, 0.10) | -0.01 (-0.10, 0.09) |  | LIPA | -0.01 (-0.10, 0.09) | -0.01 (-0.10, 0.08) | -0.02 (-0.11, 0.07) | -0.03 (-0.13, 0.06) | -0.05 (-0.15, 0.04) |
| Sleep | 0.01 (-0.08, 0.11) | 0.02 (-0.07, 0.12) | 0.04 (-0.05, 0.14) | 0.06 (-0.03, 0.16) | 0.08 (-0.01, 0.18) |  | Sleep | -0.01 (-0.10, 0.08) | -0.02 (-0.11, 0.07) | -0.04 (-0.14, 0.05) | -0.06 (-0.16, 0.03) |  |
|  | | | | | | | | | | | | |
| **MALES (n = 2,931)** | | | | | | | | | | | | |
| Remove time from: | Add time to SB (minutes) | | | | |  | Add time to: | Remove time from SB (minutes) | | | | |
|  | 15 | 30 | 60 | 90 | 120 |  |  | 15 | 30 | 60 | 90 | 120 |
| MVPA | 0.02 (-0.11, 0.15) | 0.27 (0.14, 0.40) |  |  |  |  | MVPA | -0.02 (-0.15, 0.11) | -0.04 (-0.17, 0.09) | -0.07 (-0.20, 0.06) |  |  |
| LIPA | 0.01 (-0.13, 0.13) | 0.01 (-0.13, 0.13) | 0.01 (-0.13, 0.13) | 0.01 (-0.13, 0.13) | -0.01 (-0.14, 0.12) |  | LIPA | -0.01 (-0.13, 0.13) | -0.01 (-0.14, 0.12) | -0.02 (-0.15, 0.11) | -0.03 (-0.16, 0.10) | -0.05 (-0.18, 0.08) |
| Sleep | 0.01 (-0.12, 0.14) | 0.03 (-0.10, 0.16) | 0.05 (-0.08, 0.18) | 0.08 (-0.06, 0.21) | 0.10 (-0.03, 0.23) |  | Sleep | -0.01 (-0.14, 0.12) | -0.02 (-0.15, 0.11) | -0.05 (-0.18, 0.08) | -0.07 (-0.20, 0.06) |  |
|  | | | | | | | | | | | | |
| **FEMALES (n = 2,712)** | | | | | | | | | | | | |
| Remove time from: | Add time to SB (minutes) | | | | |  | Add time to: | Remove time from SB (minutes) | | | | |
|  | 15 | 30 | 60 | 90 | 120 |  |  | 15 | 30 | 60 | 90 | 120 |
| MVPA | 0.03 (-0.11, 0.17) | 0.26 (0.12, 0.40) |  |  |  |  | MVPA | -0.03 (-0.17, 0.11) | -0.05 (-0.19, 0.09) | -0.08 (-0.22, 0.06) |  |  |
| LIPA | 0.01 (-0.14, 0.14) | 0.01 (-0.14, 0.14) | 0.01 (-0.15, 0.13) | 0.01 (-0.15, 0.13) | 0.01 (-0.14, 0.14) |  | LIPA | -0.01 (-0.14, 0.14) | -0.01 (-0.15, 0.13) | -0.02 (-0.16, 0.12) | -0.03 (-0.17, 0.11) | -0.05 (-0.19, 0.09) |
| Sleep | 0.01 (-0.13, 0.15) | 0.02 (-0.12, 0.16) | 0.03 (-0.11, 0.17) | 0.05 (-0.10, 0.19) | 0.06 (-0.08, 0.20) |  | Sleep | -0.01 (-0.15, 0.13) | -0.02 (-0.16, 0.12) | -0.03 (-0.17, 0.11) | -0.05 (-0.19, 0.09) |  |

*Note*: Data presented as estimated points change in CES-D score (95% confidence interval). All estimates adjusted for age, sex, race, employment status, education level, marital status, smoking status, alcohol consumption, diet quality score, comorbidity burden score, baseline depression symptoms. Values reflect estimated points change in CES-D score with re-allocating time from the movement behaviour in the column to the movement behaviour in the row using the mean movement behaviour composition as the reference. For example, replacing 30-minutes of SB with 30-minutes of MVPA would result in a decrease of 0.04 points in depression symptoms in the full sample. Lower CES-D score indicates less depression symptoms, higher score indicates more depression symptoms. MVPA moderate-to-vigorous physical activity, LIPA light-intensity physical activity, SB sedentary behaviour.

**Supplementary Table 4.** Estimates for depression symptoms as measured by CES-D score scale when displacing time into or out of sleep.

*Note*: Data presented as estimated points change in CES-D score (95% confidence interval). All estimates adjusted for age, sex, race, employment status, education level, marital status, smoking status, alcohol consumption, diet quality score, comorbidity burden score, baseline depression symptoms. Values reflect estimated points change in CES-D score with re-allocating time from the movement behaviour in the column to the movement behaviour in the row using the mean movement behaviour composition as the reference. For example, replacing 30-minutes of sleep with 30-minutes of MVPA would result in an increase of 0.01 points in depression symptoms in the full sample. Lower CES-D score indicates less depression symptoms, higher score indicates more depression symptoms. MVPA moderate-to-vigorous physical activity, LIPA light-intensity physical activity, SB sedentary behaviour.

| **FULL SAMPLE (n = 5,643)** | | | | | | | | | | | | |
| --- | --- | --- | --- | --- | --- | --- | --- | --- | --- | --- | --- | --- |
| Remove time from: | Add time to sleep (minutes) | | | | |  | Add time to: | Remove time from sleep (minutes) | | | | |
|  | 15 | 30 | 60 | 90 | 120 |  |  | 15 | 30 | 60 | 90 | 120 |
| MVPA | 0.01 (-0.08, 0.11) | 0.22 (0.13, 0.32) |  |  |  |  | MVPA | 0.01 (-0.09, 0.10) | 0.01 (-0.09, 0.10) | 0.02 (-0.08, 0.11) |  |  |
| LIPA | -0.01 (-0.10, 0.09) | -0.01 (-0.11, 0.08) | -0.03 (-0.12, 0.07) | -0.05 (-0.14, 0.05) |  |  | LIPA | 0.01 (-0.09, 0.10) | 0.02 (-0.08, 0.11) | 0.03 (-0.06, 0.13) | 0.05 (-0.05, 0.14) | 0.06 (-0.03, 0.16) |
| SB | -0.01 (-0.10, 0.08) | -0.02 (-0.11, 0.07) | -0.04 (-0.14, 0.05) | -0.06 (-0.16, 0.03) |  |  | SB | 0.01 (-0.08, 0.11) | 0.02 (-0.07, 0.12) | 0.04 (-0.05, 0.14) | 0.06 (-0.03, 0.16) | 0.08 (-0.01, 0.18) |
|  | | | | | | | | | | | | |
| **MALES (n = 2,931)** | | | | | | | | | | | | |
| Remove time from: | Add time to sleep (minutes) | | | | |  | Add time to: | Remove time from sleep (minutes) | | | | |
|  | 15 | 30 | 60 | 90 | 120 |  |  | 15 | 30 | 60 | 90 | 120 |
| MVPA | 0.01 (-0.12, 0.14) | 0.24 (0.11, 0.37) |  |  |  |  | MVPA | 0.01 (-0.12, 0.14) | 0.01 (-0.12, 0.15) | 0.03 (-0.10, 0.16) |  |  |
| LIPA | -0.01 (-0.14, 0.12) | -0.02 (-0.15, 0.11) | -0.04 (-0.17, 0.09) | -0.06 (-0.19, 0.07) |  |  | LIPA | 0.01 (-0.12, 0.14) | 0.02 (-0.11, 0.15) | 0.04 (-0.09, 0.17) | 0.06 (-0.07, 0.19) | 0.08 (-0.05, 0.21) |
| SB | -0.01 (-0.14, 0.12) | -0.02 (-0.15, 0.11) | -0.04 (-0.18, 0.08) | -0.07 (-0.20, 0.06) |  |  | SB | 0.01 (-0.12, 0.14) | 0.03 (-0.10, 0.16) | 0.05 (-0.08, 0.18) | 0.07 (-0.06, 0.21) | 0.10 (-0.03, 0.23) |
|  | | | | | | | | | | | | |
| **FEMALES (n = 2,712)** | | | | | | | | | | | | |
| Remove time from: | Add time to sleep (minutes) | | | | |  | Add time to: | Remove time from sleep (minutes) | | | | |
|  | 15 | 30 | 60 | 90 | 120 |  |  | 15 | 30 | 60 | 90 | 120 |
| MVPA | 0.02 (-0.12, 0.16) | 0.25 (0.11, 0.39) |  |  |  |  | MVPA | -0.01 (-0.15, 0.14) | -0.01 (-0.15, 0.13) | -0.01 (-0.15, 0.13) |  |  |
| LIPA | -0.01 (-0.14, 0.14) | -0.01 (-0.15, 0.13) | -0.02 (-0.16, 0.12) | -0.03 (-0.17, 0.11) |  |  | LIPA | 0.01 (-0.14, 0.15) | 0.01 (-0.13, 0.15) | 0.02 (-0.12, 0.16) | 0.03 (-0.11, 0.17) | 0.04 (-0.10, 0.18) |
| SB | -0.01 (-0.15, 0.13) | 0.02 (-0.16, 0.12) | -0.03 (-0.17, 0.11) | -0.05 (-0.19, 0.09) |  |  | SB | 0.01 (-0.13, 0.15) | 0.02 (-0.12, 0.16) | 0.03 (-0.11, 0.17) | 0.05 (-0.10, 0.19) | 0.06 (-0.08, 0.20) |

**Supplementary Table 5.** Estimated change in depression symptoms as measured by CES-D score scale when displacing time between one and remaining movement behaviours proportionally.

| **FULL SAMPLE (n = 5,643)** | | | | | | | | | | | | |
| --- | --- | --- | --- | --- | --- | --- | --- | --- | --- | --- | --- | --- |
| Remove time from: | Minutes | | | | |  | Add time to: | Minutes | | | | |
|  | 15 | 30 | 60 | 90 | 120 |  |  | 15 | 30 | 60 | 90 | 120 |
| MVPA | 0.02 (-0.08, 0.11) | 0.23 (0.14, 0.33) |  |  |  |  | MVPA | -0.01 (-0.11, 0.08) | -0.02 (-0.12, 0.07) | -0.04 (-0.14, 0.05) |  |  |
| LIPA | -0.01 (-0.10, 0.09) | -0.01 (-0.10, 0.09) | -0.02 (-0.11, 0.08) | -0.03 (-0.12, 0.07) | -0.04 (-0.14, 0.05) |  | LIPA | 0.01 (-0.09, 0.10) | 0.01 (-0.09, 0.10) | 0.01 (-0.08, 0.11) | 0.02 (-0.08, 0.11) | 0.02 (-0.07, 0.12) |
| SB | -0.01 (-0.10, 0.09) | -0.02 (-0.11, 0.08) | -0.04 (-0.13, 0.05) | -0.06 (-0.16, 0.03) | -0.09 (-0.18, 0.00) |  | SB | 0.01 (-0.09, 0.10) | 0.02 (-0.08, 0.11) | 0.04 (-0.06, 0.13) | 0.05 (-0.04, 0.15) | 0.07 (-0.02, 0.16) |
| Sleep | 0.01 (-0.08, 0.10) | 0.02 (-0.07, 0.11) | 0.04 (-0.06, 0.13) | 0.06 (-0.04, 0.15) | 0.08 (-0.02, 0.17) |  | Sleep | -0.01 (-0.10, 0.08) | -0.02 (-0.11, 0.07) | -0.04 (-0.14, 0.05) | -0.06 (-0.16, 0.03) |  |
|  | | | | | | | | | | | | |
| **MALES (n = 2,931)** | | | | | | | | | | | | |
| Remove time from: | Minutes | | | | |  | Add time to: | Minutes | | | | |
|  | 15 | 30 | 60 | 90 | 120 |  |  | 15 | 30 | 60 | 90 | 120 |
| MVPA | 0.02 (-0.11, 0.15) | 0.26 (0.13, 0.39) |  |  |  |  | MVPA | -0.01 (-0.14, 0.12) | -0.02 (-0.15, 0.11) | -0.05 (-0.18, 0.08) |  |  |
| LIPA | -0.01 (-0.13, 0.12) | -0.01 (-0.14, 0.12) | -0.02 (-0.15, 0.11) | -0.04 (-0.17, 0.09) | -0.06 (-0.19, 0.07) |  | LIPA | 0.01 (-0.12, 0.13) | 0.01 (-0.12, 0.14) | 0.02 (-0.11, 0.15) | 0.03 (-0.10, 0.16) | 0.03 (-0.10, 0.16) |
| SB | -0.01 (-0.14, 0.12) | -0.02 (-0.15, 0.11) | -0.04 (-0.17, 0.09) | -0.06 (-0.19, 0.07) | -0.09 (-0.21, 0.04) |  | SB | 0.01 (-0.12, 0.14) | 0.02 (-0.11, 0.15) | 0.03 (-0.10, 0.16) | 0.05 (-0.08, 0.18) | 0.06 (-0.07, 0.19) |
| Sleep | 0.01 (-0.12, 0.14) | 0.02 (-0.11, 0.15) | 0.05 (-0.08, 0.18) | 0.07 (-0.06, 0.20) | 0.09 (-0.03, 0.23) |  | Sleep | -0.01 (-0.14, 0.12) | -0.02 (-0.15, 0.11) | -0.05 (-0.18, 0.08) | -0.08 (-0.20, 0.05) |  |

| **FEMALES (n = 2,712)** | | | | | | | | | | | | |
| --- | --- | --- | --- | --- | --- | --- | --- | --- | --- | --- | --- | --- |
| Remove time from: | Minutes | | | | |  | Add time to: | Minutes | | | | |
|  | 15 | 30 | 60 | 90 | 120 |  |  | 15 | 30 | 60 | 90 | 120 |
| MVPA | 0.02 (-0.12, 0.16) | 0.26 (0.12, 0.40) |  |  |  |  | MVPA | -0.02 (-0.15, 0.12) | -0.03 (-0.16, 0.11) | -0.05 (-0.19, 0.09) |  |  |
| LIPA | -0.01 (-0.14, 0.14) | -0.01 (-0.15, 0.13) | -0.01 (-0.15, 0.13) | -0.02 (-0.16, 0.12) | -0.03 (-0.17, 0.11) |  | LIPA | 0.01 (-0.14, 0.14) | 0.01 (-0.14, 0.14) | 0.01 (-0.13, 0.15) | 0.01 (-0.13, 0.15) | 0.02 (-0.12, 0.16) |
| SB | -0.01 (-0.15, 0.13) | -0.02 (-0.16, 0.12) | -0.03 (-0.17, 0.11) | -0.05 (-0.19, 0.09) | -0.07 (0.21, 0.07) |  | SB | 0.01 (-0.13, 0.15) | 0.02 (-0.12, 0.16) | 0.03 (-0.11, 0.17) | 0.05 (-0.09, 0.19) | 0.06 (-0.08, 0.20) |
| Sleep | 0.01 (-0.13, 0.15) | 0.01 (-0.13, 0.15) | 0.02 (-0.12, 0.16) | 0.04 (-0.10, 0.18) | 0.05 (-0.09, 0.19) |  | Sleep | -0.01 (-0.15, 0.13) | -0.01 (-0.15, 0.13) | -0.02 (-0.16, 0.12) | -0.04 (-0.18, 0.10) |  |

*Note*: Data presented as estimated points change in CES-D score (95% confidence interval). All estimates adjusted for age, sex, race, employment status, education level, marital status, smoking status, alcohol consumption, diet quality score, comorbidity burden score, baseline depression symptoms. Values reflect estimated points change in CES-D score with re-allocating time from one behaviour to the remaining movement behaviours proportionally (or vice versa) using the mean movement behaviour composition as the reference. For example, replacing 30-minutes of MVPA with 6.25-minutes LIPA, 8.75-minutes SB and 15-minutes sleep would result in an increase of 0.23 points in depression symptoms in the full sample. Lower CES-D score indicates less depression symptoms, higher score indicates more depression symptoms. MVPA moderate-to-vigorous physical activity, LIPA light-intensity physical activity, SB sedentary behaviour.

**Table 6**. ORs for depression as measured by CES-D score scale when displacing 30-minutes between movement behaviours, full sample (n = 5,643)

| **Remove 30-min**  **per day**  **from** |  | **Add 30-min per day to** | | |
| --- | --- | --- | --- | --- |
|  | **MVPA** | **LIPA** | **SB** | **Sleep** |
| MVPA | – | 1.14 (1.13, 1.15) | 1.17 (1.16, 1.18) | 1.07 (1.06, 1.08) |
| LIPA | 0.93 (0.92, 0.94) | – | 1.06 (1.05, 1.07) | 0.95 (0.94, 0.96) |
| SB | 0.85 (0.84, 0.86) | 0.94 (0.93, 0.95) | – | 0.90 (0.89, 0.91) |
| Sleep | 0.99 (0.98, 1.00) | 1.03 (1.02, 1.04) | 1.09 (1.08, 1.10) | – |

**Table 7**. ORs for depression as measured by CES-D score scale when displacing 30-minutes between movement behaviours, males (n = 2,931)

| **Remove 30-min**  **per day**  **from** |  | **Add 30-min per day to** | | |
| --- | --- | --- | --- | --- |
|  | **MVPA** | **LIPA** | **SB** | **Sleep** |
| MVPA | – | 1.12 (1.11, 1.14) | 1.17 (1.16, 1.19) | 1.10 (1.09, 1.11) |
| LIPA | 0.94 (0.93, 0.95) | – | 1.07 (1.06, 1.08) | 1.00 (0.99, 1.01) |
| SB | 0.91 (0.90, 0.92) | 0.93 (0.92, 0.94) | – | 0.93 (0.92, 0.94) |
| Sleep | 1.02 (0.89, 1.16) | 1.00 (0.99, 1.01) | 1.07 (1.06, 1.08) | – |

**Table 8**. ORs for depression as measured by CES-D score scale when displacing 30-minutes between movement behaviours, females (n = 2,712)

| **Remove 30-min**  **per day**  **from** |  | **Add 30-min per day to** | | |
| --- | --- | --- | --- | --- |
|  | **MVPA** | **LIPA** | **SB** | **Sleep** |
| MVPA | – | 1.19 (1.17, 1.20) | 1.20 (1.19, 1.22) | 1.09 (1.08, 1.11) |
| LIPA | 0.89 (0.88, 0.90) | – | 1.04 (1.03, 1.05) | 0.92 (0.91, 0.93) |
| SB | 0.88 (0.87, 0.89) | 0.95 (0.94, 0.96) | – | 0.89 (0.88, 0.90) |
| Sleep | 0.97 (0.96, 0.98) | 1.05 (1.04, 1.07) | 1.10 (1.08, 1.11) | – |

*Note*: Data presented as OR (95% confidence interval). All estimates adjusted for age, sex, race, employment status, education level, marital status, smoking status, alcohol consumption, diet quality score, comorbidity burden score, baseline depression symptoms. Values reflect OR for depression (CES-D score of ≥10) when re-allocating 30-minutes from the movement behaviour in the column to the movement behaviour in the row using the mean movement behaviour composition as the reference. For example, in Table 6 replacing 30-minutes of MVPA with 30-minutes of LIPA would result in increased odds for depression (OR = 1.14, 95%CI: 1.13, 1.15). MVPA moderate-to-vigorous physical activity, LIPA light-intensity physical activity, SB sedentary behaviour.

**Table 9**. ORs for depression as measured by CES-D score scale when displacing 30-minutes from one movement behaviour to remaining behaviours proportionally, full sample (n = 5,643)

| **Movement behaviour** | **Remove 30-min of column**  **behaviour and add to remaining behaviours** | **Add 30-min to column behaviour**  **from remaining behaviours** |
| --- | --- | --- |
| MVPA | 1.12 (1.11, 1.13) | 0.95 (0.94, 0.96) |
| LIPA | 1.01 (1.00, 1.02) | 0.99 (0.98, 1.00) |
| SB | 0.92 (0.91, 0.93) | 1.09 (1.08, 1.10) |
| Sleep | 1.08 (1.07, 1.09) | 0.93 (0.92, 0.94) |

**Table 10**. ORs for depression as measured by CES-D score scale when displacing 30-minutes from one movement behaviour to remaining behaviours proportionally, males (n = 2,931)

| **Movement behaviour** | **Remove 30-min of column**  **behaviour and add to remaining behaviours** | **Add 30-min to column behaviour from remaining behaviours** |
| --- | --- | --- |
| MVPA | 1.13 (1.11, 1.14) | 0.94 (0.93, 0.95) |
| LIPA | 1.03 (1.02, 1.04) | 0.97 (0.96, 0.98) |
| SB | 0.92 (0.91, 0.93) | 1.08 (1.07, 1.09) |
| Sleep | 1.03 (1.02, 1.04) | 0.97 (0.96, 0.98) |

**Table 11**. ORs for depression as measured by CES-D score scale when displacing 30-minutes from one movement behaviour to remaining behaviours proportionally, females (n = 2,712)

| **Movement behaviour** | **Remove 30-min of column**  **behaviour and add to remaining behaviours** | **Add 30-min to column behaviour from remaining behaviours** |
| --- | --- | --- |
| MVPA | 1.15 (1.13, 1.16) | 0.96 (0.95, 0.97) |
| LIPA | 0.99 (0.97, 1.00) | 1.01 (1.00, 1.02) |
| SB | 0.92 (0.91, 0.93) | 1.08 (1.07, 1.10) |
| Sleep | 1.11 (1.09, 1.12) | 0.90 (0.89, 0.91) |

Note: Data presented as OR (95% confidence interval). All estimates adjusted for age, sex, race, employment status, education level, marital status, smoking status, alcohol consumption, diet quality score, comorbidity burden score, baseline depression symptoms. Values reflect OR for depression (CES-D score of ≥10) when re-allocating 30-minutes from the movement behaviour in the column to the movement behaviour in the row using the mean movement behaviour composition as the reference. For example, in Table 9 replacing 30-minutes of MVPA with 6.62-minutes LIPA, 8.53-minutes SB and 14.85-minutes sleep would result in increased odds of depression (OR= 1.12, 95%CI: 1.11, 1.13). MVPA moderate-to-vigorous physical activity, LIPA light-intensity physical activity, SB sedentary behaviour.

Figure 1. Estimated changes in depression symptoms scores associated with hypothetical time displacements for MVPA, sex stratified.


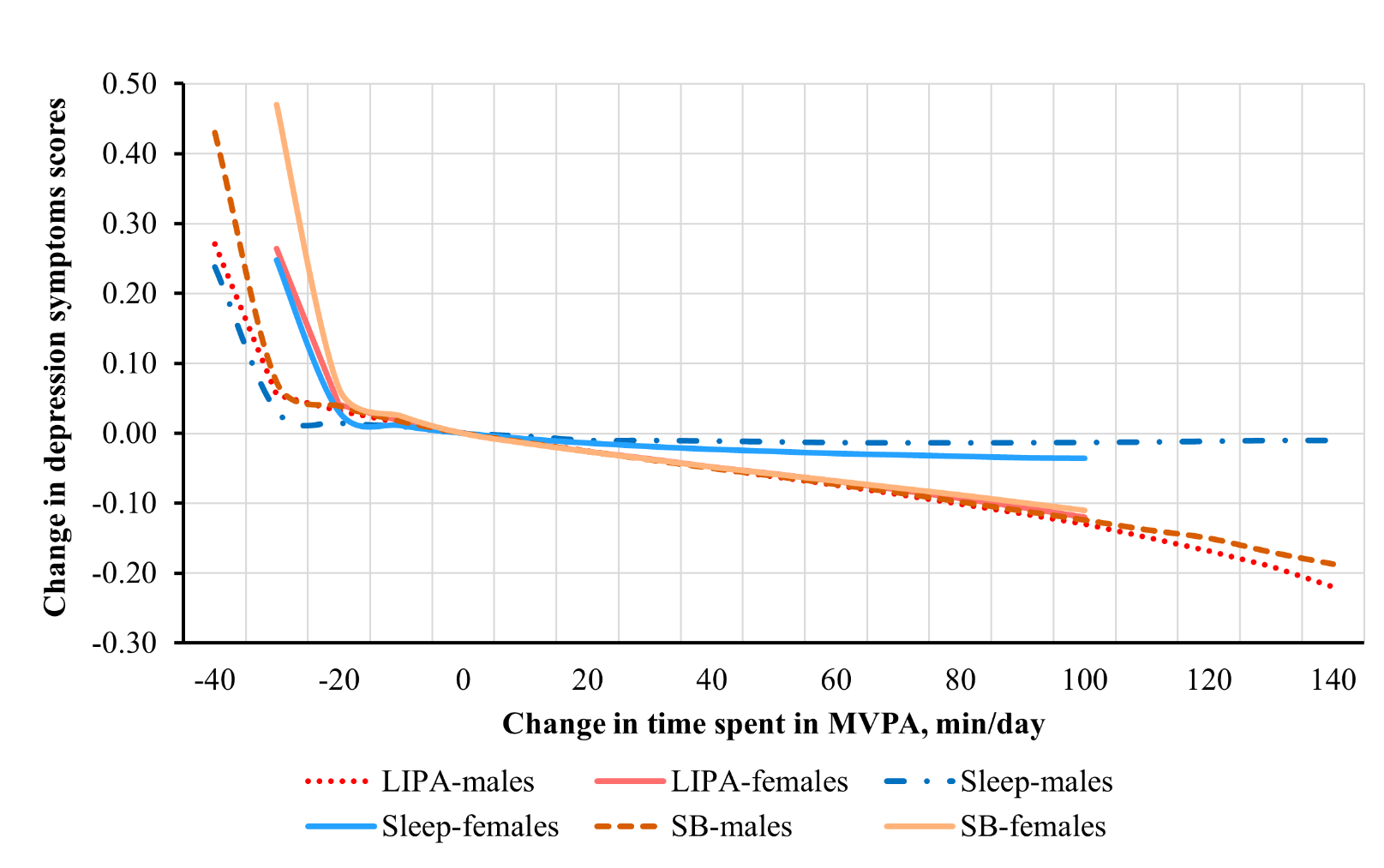


Figure 2. Estimated changes in depression symptoms scores associated with hypothetical time displacements for LIPA, sex stratified.


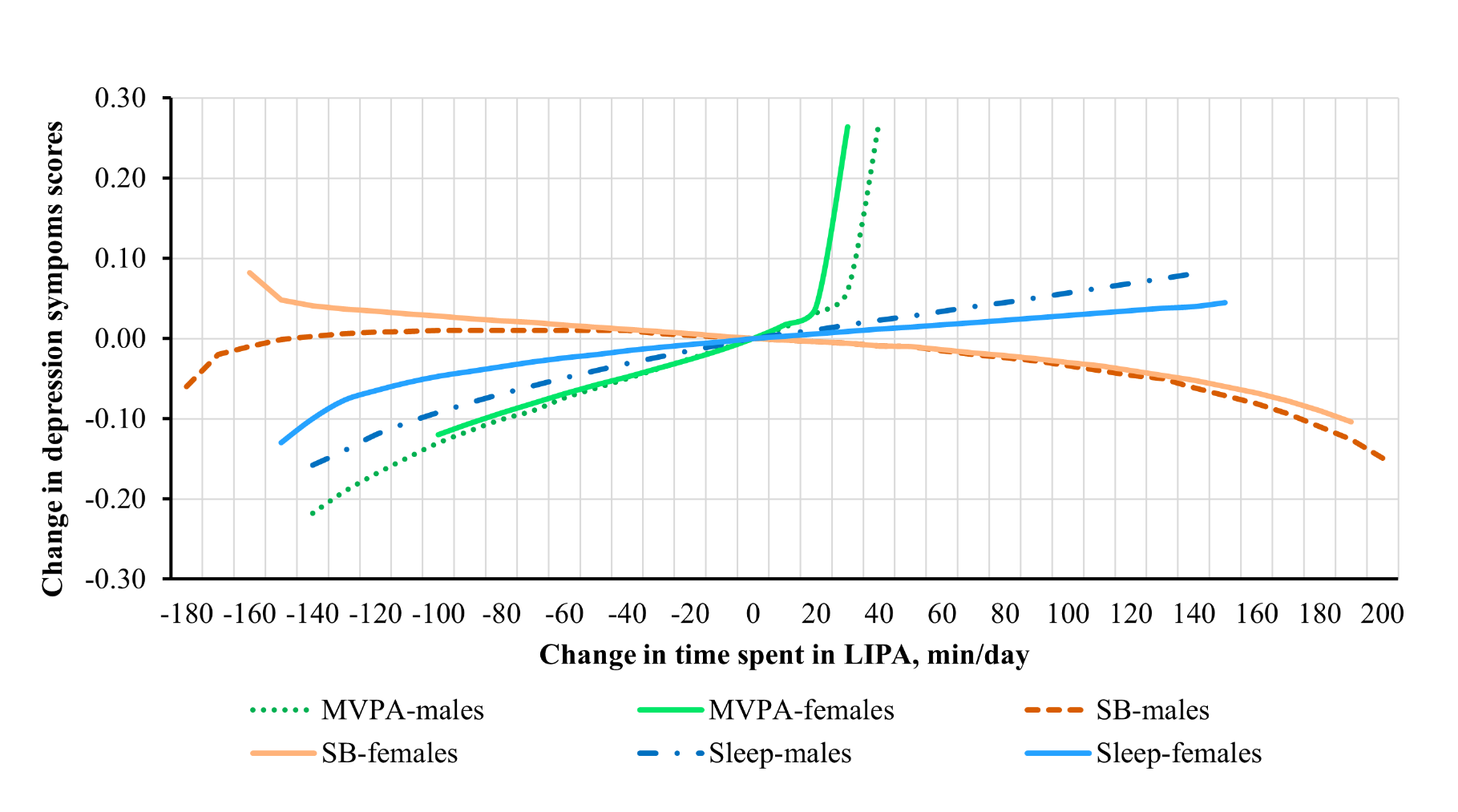


Figure 3. Estimated changes in depression symptoms scores associated with hypothetical time displacements for SB, sex stratified.


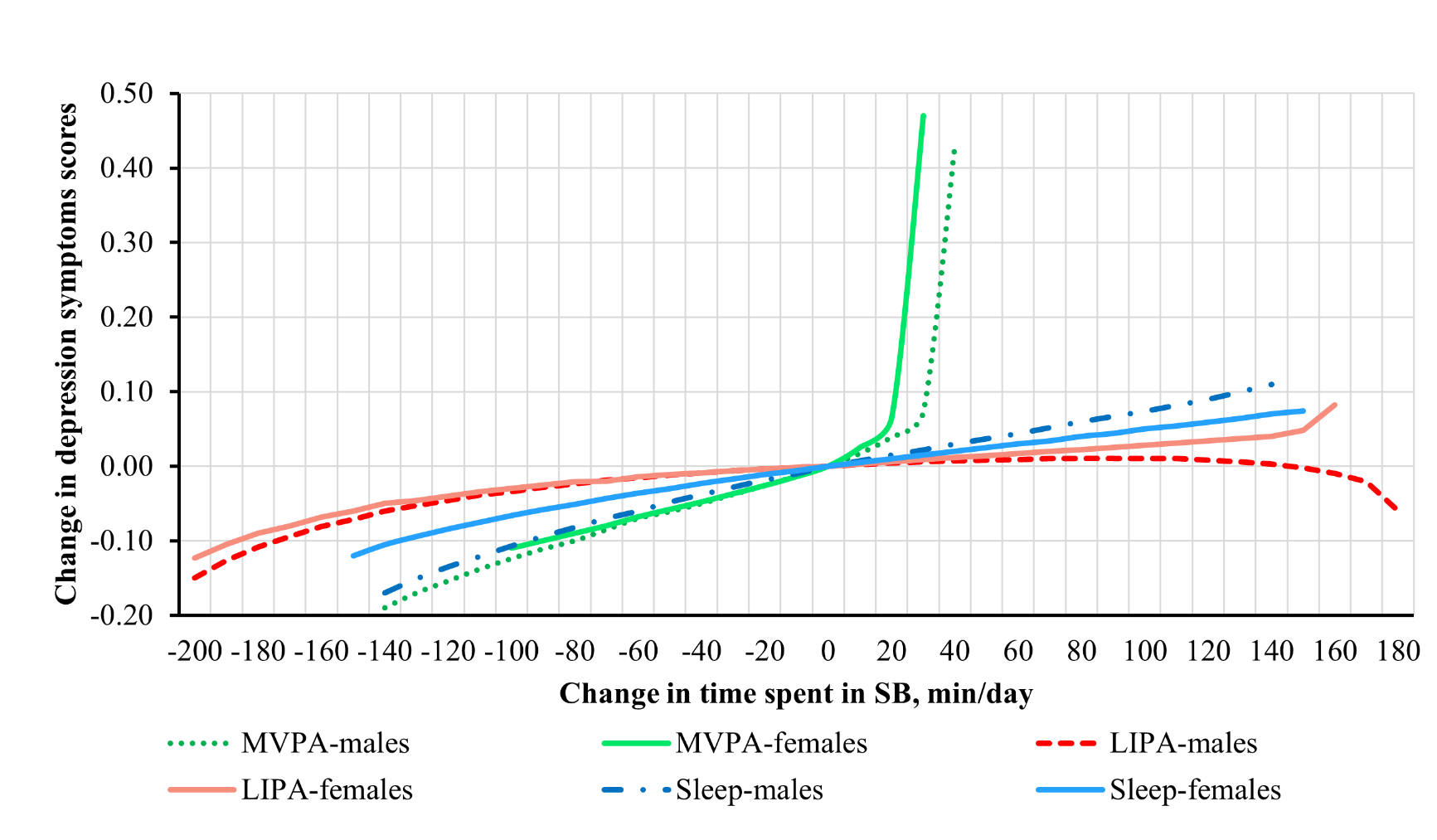


Figure 4. Estimated changes in depression symptoms scores associated with hypothetical time displacements for sleep, sex stratified.


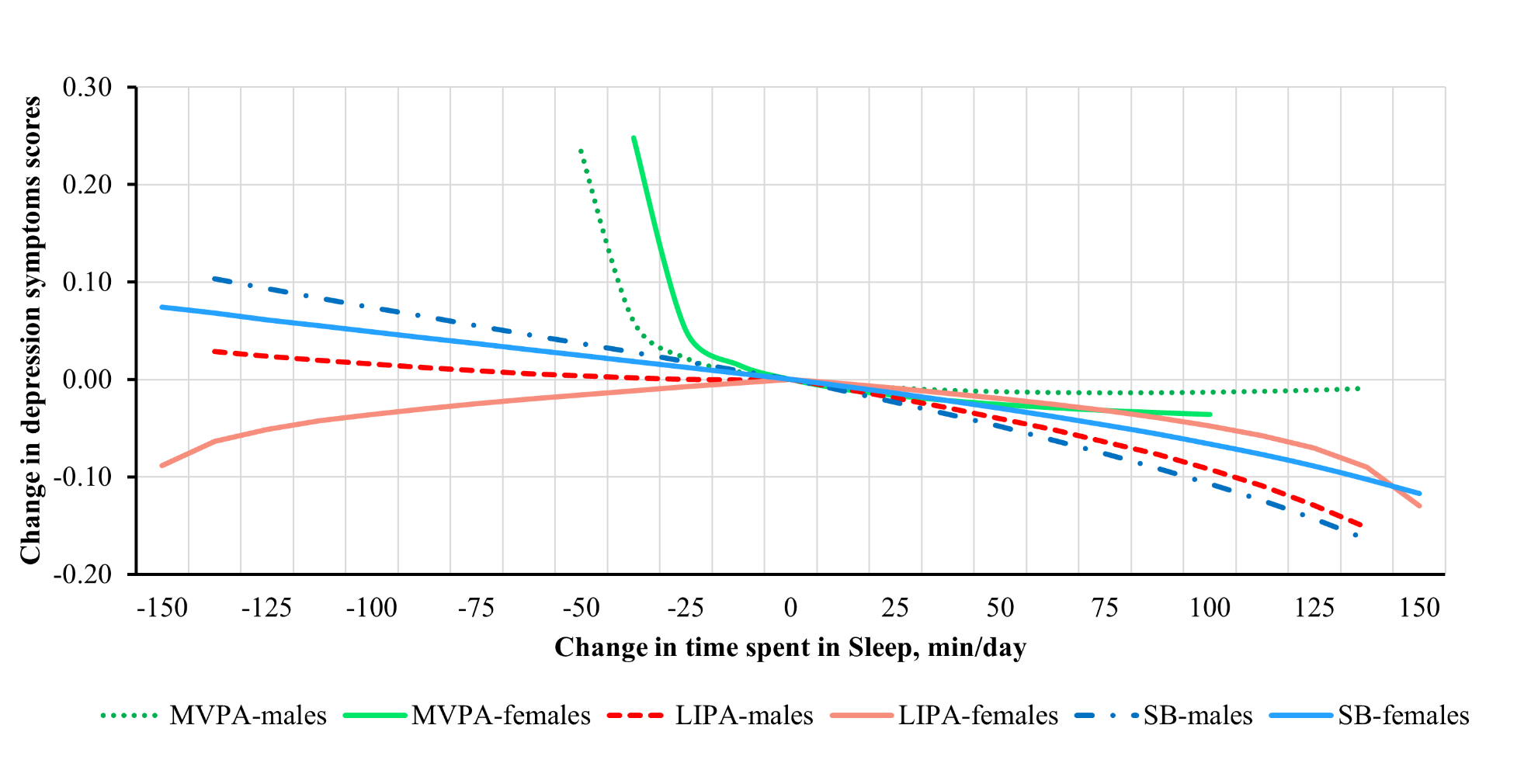


Note: For Figures 1-4 all estimates adjusted for age, sex, race, employment status, education level, marital status, smoking status, alcohol consumption, diet quality score, comorbidity burden score, baseline depression symptoms. Estimates reflect the hypothetical change in depression symptoms scores associated with reallocating time spent in each movement behaviour based on parameter estimates from compositional regression. The difference in minutes/day are modelled around the mean movement behaviour composition (reference). Time is substituted between the movement behaviour on the x-axis and the movement behaviour indicated by the line. For example, Figure 2 shows estimated scores associated with hypothetically changing the mean amount of time spent in LIPA. As more minutes are added to LIPA, it is estimated that scores will increase (higher depressive symptoms) if this time is taken from MVPA but decrease if this time is added to sleep. Substitutions were not made beyond the range of ± two standard deviations for the mean of each movement behaviour [e.g., no more than 100 min per day were added to the mean 31 min per day spent in MVPA (for females)]

Figure 5. Estimated changes in depression symptoms scores with hypothetical displacements between one and remaining movement behaviours proportionally, sex stratified.


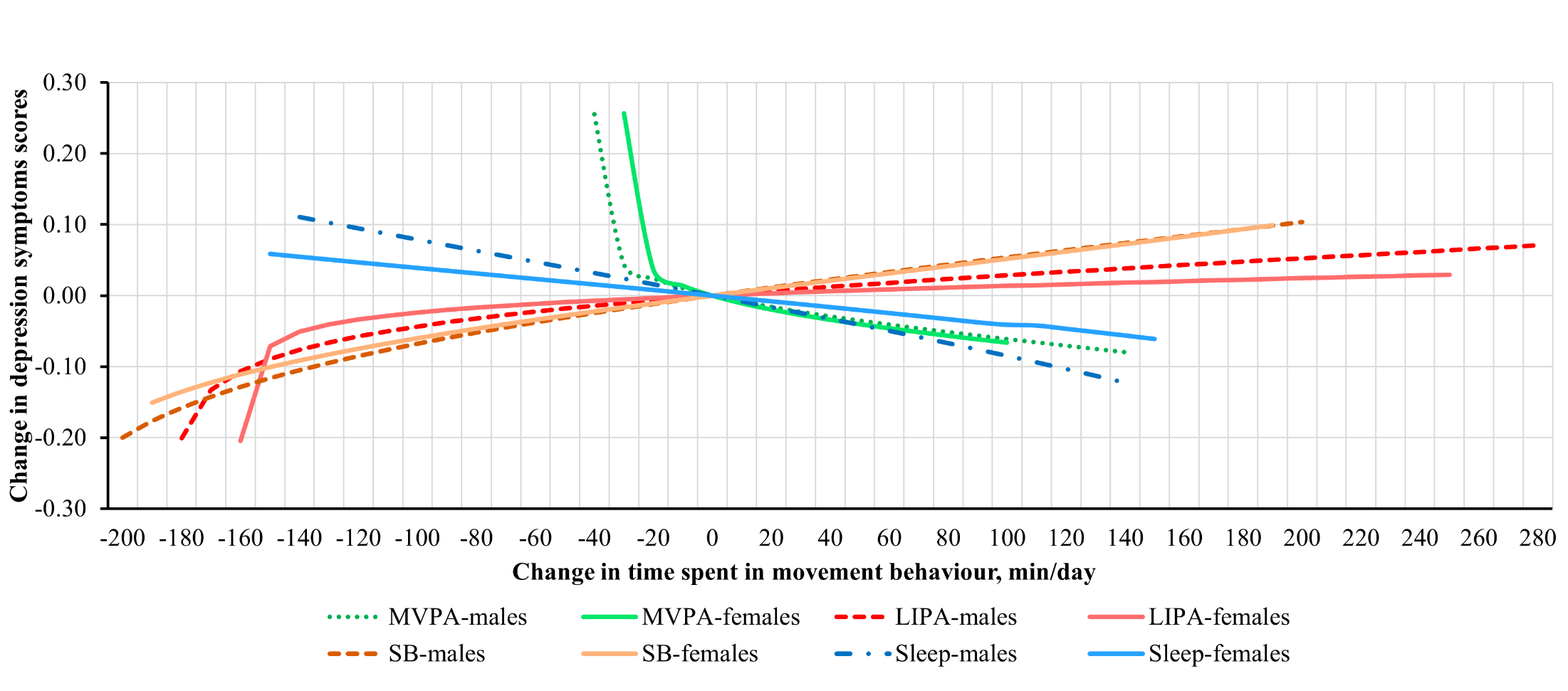


Note: All estimates adjusted for age, sex, race, employment status, education level, marital status, smoking status, alcohol consumption, diet quality score, comorbidity burden score, baseline depression symptoms. Estimates reflect the hypothetical change in depression symptoms scores associated with reallocating time between one movement behaviour and the remaining movement behaviours based on parameter estimates from compositional regression. The difference in minutes/day are modelled around the mean movement behaviour composition (reference). For example, if more time is hypothetically allocated to sleep and removed from the remaining movement behaviours, the estimated depression symptoms scores decrease. Substitutions were not made beyond the range of ± two standard deviations for the mean of each movement behaviour [e.g., no more than 100 min per day were added to the mean 31 min per day spent in MVPA (for females)]
